# Global, regional, and national temporal trend and patterns of change in the burden of leishmaniasis from 1990 to 2021: an analysis of the Global Burden of Disease Study 2021

**DOI:** 10.1016/j.soh.2025.100123

**Published:** 2025-09-06

**Authors:** Shunxian Zhang, Guobing Yang, Shan Lv, Lei Duan, Muxin Chen, Qin Liu, Liguang Tian, Shizhu Li, Jinxin Zheng

**Affiliations:** a Longhua Hospital, Shanghai University of Traditional Chinese Medicine, Shanghai 200032, China; b Gansu Provincial Center for Disease Control and Prevention, Gansu Provincial Academy of Preventive Medicine, Lanzhou 730000, Gansu, China; c National Key Laboratory of Intelligent Tracking and Forecasting for Infectious Diseases, NHC Key Laboratory of Parasite and Vector Biology, WHO Collaborating Centre for Tropical Diseases, National Center for International Research on Tropical Diseases, National Institute of Parasitic Diseases of Chinese Center for Disease Control and Prevention, Shanghai 200025, China

**Keywords:** Leishmaniasis, Frontier analysis, Decomposition analysis, Inequality analysis, Bayesian age-period-cohort

## Abstract

**Background:**

Leishmaniasis is a globally prevalent parasitic disease caused by protozoa of the genus *Leishmania*. This study utilizes Global Burden of Disease Study 2021 (GBD 2021) data to systematically evaluate the global, regional, and national epidemiological trends, thereby providing a scientific basis for optimizing the prevention and control strategies for *leishmania* infections.

**Methods:**

Data from the GBD 2021 database were analyzed to evaluate trends in age-standardized incidence rate (ASIR), age-standardized prevalence rate (ASPR), age-standardized mortality rate (ASMR), and age-standardized disability-adjusted life-years rate (ASDR) for leishmaniasis across global, regional, and national levels, stratified by age, sex, and sociodemographic index (SDI). A Bayesian age-period-cohort (BAPC) model was employed to project future burden. Analyses included the slope index of inequality and the concentration index to assess health disparities, frontier analysis to estimate achievable outcomes based on development levels, and decomposition analysis to identify the drivers of changes in DALYs number.

**Results:**

From 1990 to 2021, the ASIR of leishmaniasis decreased from 17.82 to 14.34 per 100,000 population, while the ASPR increased from 62.94 to 76.96 per 100,000 population. Most strikingly, the ASMR showed a dramatic reduction from 1.05 to 0.07 per 100,000 population. However, concerning increases were observed in absolute case numbers, with incident cases rising from 1.01 to 1.10 million and prevalent cases nearly doubling from 3.18 to 6.21 million. Notably, Syria exhibited the most severe deterioration in ASDR (average annual percentage change [AAPC] = 4.78 %, 95 % confidence interval [*CI*]: 4.67–4.89). Frontier analysis identified several high-burden countries including South Sudan, the Syrian Arab Republic, Afghanistan, Suriname, and Sudan as persistent hotspots requiring targeted interventions. A robust inverse correlation was observed between all disease metrics (ASIR, ASPR, ASMR, ASDR, and case counts) and the SDI, with all associations demonstrating high statistical significance (*P* < 0.001).

**Conclusions:**

Leishmaniasis continues to pose significant public health challenges in Southeast Asia, North Africa, and Middle East. Strengthening public health interventions, optimizing resource distribution, and focusing on health governance in low- and middle-income countries are key to addressing the ongoing burden. One Health-based integrated strategies, particularly in vector control, host management, and environmental sanitation, are essential for reducing the disease burden and achieving long-term control.

## 1 Introduction

Leishmaniasis is a parasitic disease caused by protozoa of the genus *Leishmania*. These parasites are transmitted through the bites of infected female phlebotomine sandflies, which require blood meals to develop eggs, and 70 animal species, including humans, serve as reservoirs for *Leishmania* parasites [1,2].

*Leishmania* parasites are dimorphic flagellate protozoa whose life cycle depends on alternating between two hosts. Within the sandfly vector, the parasite exists in its flagellated promastigote form. When an infected sandfly—primarily from the genus *Phlebotomus* in the Old World and *Lutzomyia* in the New World—bites a human or another host (such as dogs or rodents), the non-flagellated amastigotes present in the host are ingested into the sandfly's gut, where they transform into promastigotes and proliferate by binary fission [3,4]. Approximately 6**–**7 days after the blood meal, the sandfly migrates these newly multiplied promastigotes to its proboscis, and during subsequent feeding, inoculates them into a new host [5]. Once inside the host, the promastigotes are phagocytosed by macrophages, where they convert back to the amastigote form and multiply extensively. Infected macrophages then disseminate the parasites via lymphatic and hematogenous routes until cell rupture releases additional parasites, leading to pathological changes in organs such as the spleen, liver, bone marrow, and lymph nodes [2].

Global research on the disease burden of leishmaniasis has primarily concentrated on analyzing temporal trends in the incidence and mortality in selected countries [6,7]. Some studies have leveraged platforms such as the Global Burden of Disease (GBD) study, the Institute for Health Metrics and Evaluation (IHME), and epidemiological reports to develop preliminary estimation systems based on incidence, mortality, and disability-adjusted life years (DALYs) [[8], [9], [10]]. However, a comprehensive global assessment of leishmaniasis disease burden in recent years remains lacking. The Global Burden of Disease Study 2021 (GBD 2021) database now provides critical disease burden data at the global, regional, and national levels, enabling a more thorough evaluation of leishmaniasis epidemiological trends across different regions. Such analyses are essential to guide public health decision-makers in identifying high-priority endemic areas and in allocating resources effectively for disease control.

## 2 Methods

### 2.1 Date source

The GBD 2021 study assessed the burden of 371 diseases and injuries, along with 88 risk factors, across 204 countries and territories. Rates and counts of incidence, prevalence, mortality, and DALYs were estimated using stratified models based on age, sex, region, countries, and territories [11,12]. The analysis systematically adjusted epidemiological data to account for biases arising from variations in data sources, definitions, and measurement methods. These adjustments were implemented through sophisticated statistical models, including Bayesian priors, regularization, and trimming using the MR-BRT (stands for meta-regression with Bayesian priors, regularization, and trimming) framework, as well as DisMod-MR 2.1 (a Bayesian mixed-effects meta-regression modeling tool developed for GBD analyses), ensuring internal consistency across estimates for different regions, age groups, sexes, and time periods. The process employed standardization and calibration steps to minimize the impact of heterogeneity on study outcomes [11,12]. Mortality data in GBD 2021 primarily originated from national vital registration systems, maternal and child health surveillance networks, and census data. Incidence and prevalence data were derived from disease surveillance systems, national health surveys, and published studies [12,13]. Disability burden estimates were based on data from case notifications, hospital discharge records, household surveys, and cohort studies [12,13]. Detailed information on study design, data collection, and estimation methodologies is provided in the GBD 2021 documentation [9,14,15].

Data on leishmaniasis were retrieved from the GBD 2021 using the GBD results tool on the IHME website (http://ghdx.healthdata.org/) [12]. The dataset includes information on leishmaniasis for incidence, prevalence, mortality, and DALYs presented as both counts and rates at global, super-regional, regional (21 geographic regions), and national (204 countries and territories) levels [12].

The sociodemographic index (SDI) is a composite metric derived as the geometric mean of three standardized indicators: the total fertility rate for women under 25, the mean years of schooling for individuals aged 15 and older, and lag-distributed income per capita. SDI values range from 0 to 1 and are categorized into five groups: low (0–0.46), low-middle (0.47–0.62), middle (0.62–0.71), high-middle (0.71–0.81), and high (0.81–1.00). This classification system facilitates a systematic evaluation of how socioeconomic development levels influence health outcomes [11].

### 2.2 Statistical analysis

The disease burden of leishmaniasis, was evaluated by examining rates and absolute case counts for incidence, prevalence, mortality, and DALYs. Rates, expressed as per 100,000 population, quantify the relative burden, whereas case counts indicate the absolute burden. Both metrics are accompanied by 95 % uncertainty intervals (*UI*s).

All statistical analyses were performed using *R* software (version 4.4.1; R Foundation for Statistical Computing, Vienna, Austria; https://cran.r-project.org). Detailed descriptions of the analytical methods employed—including the estimated annual percentage change (EAPC), average annual percentage change (AAPC), and Bayesian age-period-cohort (BAPC) models—are provided in subsequent sections [[15], [16], [17], [18]].

#### 2.2.1 EAPC

To assess the temporal trends of leishmaniasis in 1990–2021 and 2022–2035, EAPC was employed to quantify variations in key disease burden metrics—namely, the age-standardized incidence rate (ASIR), age-standardized prevalence rate (ASPR), age-standardized mortality rate (ASMR), and age-standardized DALY rate (ASDR). The EAPC was calculated using a log-linear regression model defined as [16]:

y=α+βx

where *y* is equal to natural logarithm of (rate), *x* signifies the calendar year. A statistically significant upward trend is defined by an EAPC greater than 0 with a lower 95 % confidence limit above 0. Conversely, a significant downward trend is defined by an EAPC less than 0 with an upper 95 % confidence limit below 0.

#### 2.2.2 AAPC

Joinpoint regression analysis was employed to examine temporal trends in burden metrics—including ASIR, ASPR, ASMR, and ASDR—for leishmaniasis. This analysis utilized segmented regression applied to the log-linear model, ln(*y*) = *β* × year + constant, to identify inflection points in the trends [16,19].

$${\text{APC}}_{i}=\left\{\exp\left(\beta_{i}\right)-1\right\}\times 100$$

$${\text{AAPC}}_{i}=\left\{\exp\left(\frac{\sum W_{i}\beta_{i}}{\sum W_{i}}\right)-1\right\}\times 100$$

In the model, *i* is the number of segments, *β*_*i*_ is the regression coefficient from the log-linear model ln(*y*) = *β* × year + constant. *W*_*i*_ represented the length of each corresponding segment. When the AAPC is greater than 0 with a *P*-value of less than 0.05, it indicates a statistically significant upward trend. Conversely, an AAPC less than 0 with a *P-*value below 0.05 denotes a statistically significant downward trend [16].

#### 2.2.3 BAPC

This study employed the BAPC model to project disease burden metrics (ASIR, ASPR, ASMR, and ASDR) from 2022 to 2035, leveraging its effectiveness and robustness for long-term predictions. In this analysis, we utilized the “BAPC” *R* package, incorporating data from GBD 2021 and population projections from IHME, to forecast the global burden of leishmaniasis [12,16]. A key advantage of the BAPC model is its use of the Integrated Nested Laplace Approximation method to approximate marginal posterior distributions [16]. The calculation formula is as follows [20,21]:

$$\log\left(\lambda_{ij}\right)=\alpha +\mu_{i}+\beta_{j}+\gamma_{k}$$

In this model, *i* (1 ≤ *i* ≤ *I*) represents time points, *j* (1 ≤ *j* ≤ *J*) denotes age groups, α represents the intercept, *μ*_*i*_ represents the age effect, *β*_*j*_ represents the period effect, *γ*_*k*_ represents the cohort effect. This approach effectively circumvents challenges such as mixing and convergence issues commonly associated with Markov Chain Monte Carlo techniques, while maintaining computational efficiency [12,16]. The model's flexibility and precision make it particularly effective for analyzing time-series data in epidemiological contexts.

### 2.3 Decomposition analysis

The Das Gupta decomposition method was employed to analyze the changes in the DALYs cases of leishmaniasis from 1990 to 2021. This method decomposes the overall disease burden change into three components: aging, population growth, and epidemiological changes. By breaking down the disease burden variations, the method clarifies how demographic and epidemiological shifts contribute to these trends. Unlike traditional methods, such as linear regression, which focus on exploring relationships between variables, decomposition analysis offers a detailed assessment of the independent contribution of each factor to the change in disease burden. This approach provides a deeper understanding of the core drivers behind the changes in leishmaniasis DALYs cases [16].

### 2.4 Cross-country health inequality analysis

The study quantifies both the absolute and relative inequalities in leishmaniasis ASDR using the slope index of inequality (SII) and the concentration index (CCI), as defined by the World Health Organization. The SII is computed by regressing the ASDR against the SDI, using the midpoint of the cumulative population distribution sorted by SDI. To control bias and heterogeneity, a robust regression model is used instead of the standard linear regression model in health inequality analysis. Additionally, the CCI is calculated by matching the cumulative proportion of ASDR with the cumulative population distribution ranked by SDI, followed by numerical integration of the area under the Lorenz curve [16].

### 2.5 Frontier analysis

To evaluate the relationship between the disease burden of leishmaniasis and SDI, frontier analysis was conducted using an ASDR-based model and the SDI. Specifically, locally weighted regression combined with local polynomial regression was applied, using different smoothing spans (0.3, 0.4, and 0.5) to generate a smooth frontier line. This method accurately captures the non-linear relationship between SDI and leishmaniasis ASDR. To ensure robustness, a bootstrap sampling of 1000 iterations was performed to calculate the average ASDR for each SDI value. By measuring the absolute distance (effective difference) between each country's or region's 2021 ASDR and the frontier line, we assessed the potential for improvement [16].

## 3 Results

### 3.1 Global

From 1990 to 2021, the ASIR decreased from 17.82 per 100,000 population (95 % *UI*: 15.14–21.05 per 100,000 population) to 14.34 per 100,000 population (95 % *UI*: 12.66–16.19 per 100,000 population), while the ASPR increased from 62.94 per 100,000 population (95 % *UI*: 52.92–76.63 per 100,000 population) to 76.96 per 100,000 population (95 % *UI*: 71.89–82.99 per 100,000 population). The ASMR dropped from 1.05 per 100,000 population (95 % *UI*: 0.34–3.36 per 100,000 population) to 0.07 per 100,000 population (95 % *UI*: 0.02–0.24 per 100,000 population), and the ASDR decreased from 79.59 per 100,000 population (95 % *UI*: 29.90–246.55 per 100,000 population) to 10.27 per 100,000 population (95 % *UI*: 5.99–21.96 per 100,000 population) (Table 1, Table 2, Table 3, Table 4). In addition, the global incident cases increased from 1.01 million in 1990 (95 % *UI*: 0.86–1.19 million) to 1.10 million in 2021 (95 % *UI*: 0.97–1.24 million). Additionally, the prevalent cases also showed a significant increase, rising from 3.18 million in 1990 (95 % *UI*: 2.66–3.88 million) to 6.21 million in 2021 (95 % *UI*: 5.83–6.69 million). However, the number of deaths decreased from 0.06 million in 1990 (95 % *UI*: 0.02–0.19 million) to 0.01 million in 2021 (95 % *UI*: 0.01–0.02 million). Regarding DALY cases, they decreased from 4.67 million in 1990 (95 % *UI*: 1.75–14.55 million) to 0.78 million in 2021 (95 % *UI*: 0.47–1.64 million) (Table S1–S4).

**Table 1:** The ASIR of leishmaniasis in 1990 and 2021 and its temporal trends across 21 GBD geographical regions.

Location	ASIR (per 100,000 population) (95 % *UI*), 1990	ASIR (per 100,000 population) (95 % *UI*), 2021	PC (95 % *UI*), 1990–2021	EAPC (95 % *CI*), 1990–2021	AAPC (95 % *CI*), 1990–2021
Global	17.82 (15.14**–**21.05)	14.34 (12.66**–**16.19)	−19.57 (−33.60, −1.74)	0.51 (−0.08, 1.11)	−0.12 (−0.14, −0.11)
East Asia	0.79 (0.54**–**1.27)	0.15 (0.10**–**0.25)	−80.69 (−89.67, −61.34)	−4.25 (−5.09, −3.40)	−0.02 (−0.02, −0.02)
Southeast Asia	0.18 (0.10**–**0.33)	1.16 (0.88**–**1.47)	525.67 (236.86**–**1054.71)	7.48 (5.74**–**9.26)	0.04 (0.03**–**0.05)
Central Asia	28.60 (21.44**–**37.83)	7.16 (5.33**–**9.54)	−74.98 (−82.94, −62.45)	−2.67 (−4.21, −1.11)	−0.68 (−0.71, −0.65)
Central Europe	1.64 (0.99**–**3.14)	0.16 (0.12**–**0.20)	−90.46 (−94.98, −82.45)	−8.52 (−9.16, −7.87)	−0.05 (−0.05, −0.05)
Western Europe	0.44 (0.35**–**0.58)	0.30 (0.24**–**0.38)	−31.88 (−51.81, −3.66)	−0.89 (−1.17, −0.60)	−0.01 (−0.02, −0.01)
Southern Latin America	1.95 (0.81**–**3.96)	1.78 (1.07**–**2.65)	−8.55 (−61.58, 153.62)	0.12 (−0.39, 0.63)	0.01 (−0.01, 0.02)
High-income North America	0.02 (0.01**–**0.06)	0.03 (0.01**–**0.09)	33.45 (−72.33, 685.85)	2.19 (1.74**–**2.64)	0.02 (−0.01, 0.02)
Caribbean	7.40 (3.99**–**13.05)	4.08 (2.38**–**7.15)	−44.90 (−72.98, 35.27)	−0.78 (−1.28, −0.27)	−0.12 (−0.15, −0.08)
Andean Latin America	84.48 (60.14**–**117.48)	63.08 (50.48**–**79.15)	−25.33 (−49.06, 10.73)	−1.81 (−2.17, −1.44)	−0.74 (−0.81, −0.66)
Central Latin America	38.95 (27.75**–**57.33)	27.73 (22.37**–**35.12)	−28.80 (−52.01, 7.23)	0.33 (−0.74, 1.40)	−0.27 (−0.40, −0.13)
Tropical Latin America	45.15 (35.15**–**57.74)	29.42 (24.96**–**34.57)	−34.83 (−53.18, −10.96)	−1.45 (−2.00, −0.90)	−0.54 (−0.57, −0.50)
North Africa and Middle East	101.87 (76.98**–**136.97)	130.35 (109.95**–**150.97)	27.96 (−8.51, 78.43)	1.87 (1.33**–**2.42)	1.08 (0.99**–**1.16)
South Asia	21.77 (15.35**–**29.37)	3.55 (2.23**–**5.47)	−83.67 (−90.41, −71.57)	−4.87 (−5.72, −4.00)	−0.58 (−0.61, −0.55)
Central Sub-Saharan Africa	34.97 (12.65**–**85.67)	3.54 (1.77**–**6.83)	−89.87 (−96.71, −70.51)	−3.11 (−5.07, −1.10)	−1.02 (−1.04, −0.99)
Eastern Sub-Saharan Africa	51.48 (31.60**–**79.71)	3.24 (2.65**–**3.93)	−93.70 (−96.25, −89.38)	−6.41 (−7.92, −4.88)	−1.53 (−1.55, −1.51)
Southern Sub-Saharan Africa	0.10 (0.04**–**0.20)	0.08 (0.04**–**0.16)	−18.94 (−69.31, 170.89)	0.03 (−0.45, 0.51)	−0.02 (−0.03, −0.01)
Western Sub-Saharan Africa	3.94 (2.46**–**6.26)	3.59 (2.67**–**4.75)	−9.05 (−44.57, 52.10)	−0.93 (−1.48, −0.37)	−0.02 (−0.03, −0.01)
High-middle SDI	2.78 (1.79**–**4.37)	2.26 (1.44**–**3.47)	−18.78 (−56.46, 48.72)	0.28 (−0.76, 1.33)	−0.03 (−0.04, −0.01)
High SDI	2.88 (1.27**–**5.56)	0.58 (0.29**–**1.08)	−79.87 (−91.68, −45.63)	−4.72 (−5.57, −3.85)	−0.08 (−0.09, −0.06)
Low-middle SDI	24.43 (19.48**–**30.54)	10.18 (8.36**–**12.47)	−58.34 (−70.17, −44.73)	−2.37 (−2.75, −1.99)	−0.45 (−0.46, −0.44)
Low SDI	61.41 (46.48**–**79.02)	24.83 (18.14**–**33.51)	−59.56 (−72.67, −38.55)	−0.52 (−1.71, 0.69)	−1.21 (−1.29, −1.13)
Middle SDI	15.41 (11.64**–**21.81)	24.54 (21.22**–**28.67)	59.28 (12.03**–**121.73)	2.23 (1.75**–**2.71)	0.31 (0.29**–**0.32)

Notes: The AAPC was ultimately utilized as the core statistical measure for evaluating trends in disease burden indicator. In the 21 GBD geographical regions, no data on the incidence rate of leishmaniasis have been reported from Oceania, Eastern Europe, high-income Asia Pacific, or Australasia from 1990 to 2021. Abbreviations: AAPC, average annual percent change; ASIR, age-standardized incidence rate; *CI*, confidence interval; EAPC, estimated annual percentage change; GBD, Global Burden of Disease; PC, percentage change; SDI, sociodemographic index; *UI*, uncertainty interval.

**Table 2:** The ASPR of leishmaniasis in 1990 and 2021 and its temporal trends across 21 GBD geographical regions.

Location	ASPR (per 100,000 population) (95 % *UI*), 1990	ASPR (per 100,000 population) (95 % *UI*), 2021	PC (95 % *UI*), 1990–2021	EAPC (95 % *CI*), 1990–2021	AAPC (95 % *CI*), 1990–2021
Global	62.94 (52.92**–**76.63)	76.96 (71.89**–**82.99)	22.29 (5.77**–**40.46)	0.71 (0.66**–**0.77)	0.44 (0.43**–**0.46)
East Asia	2.43 (1.08**–**5.72)	1.07 (0.59**–**2.20)	−56.00 (−63.70, −38.07)	−2.86 (−2.94, −2.78)	−0.03 (−0.04, −0.02)
Southeast Asia	1.10 (0.55**–**1.96)	2.10 (1.79**–**2.43)	91.11 (17.09**–**250.82)	2.35 (1.72**–**2.98)	0.04 (0.03**–**0.05)
Central Asia	218.92 (168.59**–**288.81)	111.87 (93.49**–**135.47)	−48.90 (−54.12, −41.73)	−2.51 (−2.61, −2.41)	−3.49 (−3.52, −3.45)
Central Europe	1.36 (0.97**–**1.94)	0.67 (0.55**–**0.83)	−50.58 (−61.09, −35.81)	−2.45 (−2.55, −2.36)	−0.02 (−0.03, −0.01)
Western Europe	0.99 (0.60**–**1.66)	0.75 (0.60**–**0.97)	−23.80 (−41.42, 6.88)	−0.93 (−0.99, −0.87)	−0.02 (−0.03, −0.01)
Southern Latin America	13.02 (5.16**–**28.55)	12.85 (10.32**–**17.45)	−1.30 (−39.88, 106.25)	0.00 (−0.25, 0.26)	−0.02 (−0.02, 0.01)
High-income North America	0.09 (0.02**–**0.23)	0.10 (0.06**–**0.16)	10.72 (−41.49, 254.97)	0.44 (0.37**–**0.50)	0.02 (−0.01, 0.03)
Caribbean	55.31 (30.04**–**97.56)	52.92 (43.21**–**66.17)	−4.31 (−31.48, 51.00)	−0.13 (−0.18, −0.08)	−0.07 (−0.09, −0.06)
Andean Latin America	551.91 (389.38**–**778.49)	548.72 (487.32**–**619.84)	−0.58 (−22.68, 30.77)	−0.14 (−0.31, 0.02)	−0.25 (−0.38, −0.13)
Central Latin America	241.10 (170.49**–**351.89)	215.86 (193.17**–**249.71)	−10.47 (−29.52, 16.11)	−0.20 (−0.32, −0.08)	−0.83 (−0.89, −0.77)
Tropical Latin America	302.62 (234.49**–**390.31)	297.45 (269.82**–**330.72)	−1.71 (−18.16, 19.90)	−0.10 (−0.29, 0.08)	−0.19 (−0.25, −0.13)
North Africa and Middle East	645.66 (480.84**–**883.73)	642.90 (592.06**–**712.93)	−0.43 (−20.32, 26.45)	0.03 (−0.06, 0.11)	−0.16 (−0.29, −0.04)
South Asia	23.78 (15.20**–**35.41)	18.86 (16.21**–**22.61)	−20.70 (−41.19, 12.24)	−0.78 (−0.99, −0.57)	−0.15 (−0.16, −0.14)
Central Sub-Saharan Africa	27.28 (13.47**–**50.12)	16.16 (12.26**–**22.26)	−40.74 (−61.02, 7.15)	−0.98 (−1.25, −0.71)	−0.36 (−0.36, −0.35)
Eastern Sub-Saharan Africa	25.76 (17.05**–**36.83)	11.00 (9.20**–**13.59)	−57.27 (−67.10, −42.89)	−2.07 (−2.46, −1.68)	−0.47 (−0.48, −0.46)
Southern Sub-Saharan Africa	0.73 (0.31**–**1.45)	0.66 (0.50**–**0.87)	−9.91 (−42.25, 73.11)	−0.23 (−0.33, −0.13)	−0.02 (−0.03, −0.01)
Western Sub-Saharan Africa	35.65 (22.12**–**55.13)	41.88 (36.63**–**48.37)	17.47 (−14.43, 72.39)	0.63 (0.37**–**0.89)	0.21 (0.20**–**0.21)
High SDI	11.79 (4.99**–**23.45)	13.93 (10.40**–**19.31)	18.12 (−21.17, 129.26)	0.36 (0.23**–**0.49)	0.07 (0.07**–**0.09)
High-middle SDI	11.96 (6.93**–**21.06)	11.91 (10.00**–**14.92)	−0.40 (−30.62, 53.82)	0.01 (−0.11, 0.12)	−0.03 (−0.05, −0.01)
Middle SDI	84.02 (63.27**–**116.97)	106.40 (99.00**–**117.23)	26.64 (−0.99, 59.36)	0.86 (0.76**–**0.96)	0.66 (0.63**–**0.70)
Low-middle SDI	136.16 (102.60**–**192.53)	99.68 (88.71**–**115.88)	−26.79 (−39.91, −12.45)	−1.18 (−1.27, −1.09)	−1.20 (−1.22, −1.17)
Low SDI	98.34 (66.25**–**146.40)	139.66 (125.52**–**156.55)	42.02 (1.60**–**97.52)	1.74 (1.45**–**2.04)	1.42 (1.35**–**1.49)

Notes: The AAPC was ultimately utilized as the core statistical measure for evaluating trends in disease burden indicator. In the 21 GBD geographical regions, no data on the prevalence rates of leishmaniasis have been reported from Oceania, Eastern Europe, high-income Asia Pacific, or Australasia from 1990 to 2021. Abbreviations: AAPC, Average annual percent change; ASPR, age-standardized prevalence rate; *CI*, confidence interval; EAPC, estimated annual percentage change; GBD, Global Burden of Disease; PC, percentage change; SDI, sociodemographic index; *UI*, uncertainty interval.

**Table 3:** The ASMR of leishmaniasis in 1990 and 2021 and its temporal trends across 21 GBD geographical regions.

Location	ASMR (per 100,000 population) (95 % *UI*), 1990	ASMR (per 100,000 population) (95 % *UI*), 2021	PC (95 % *UI*), 1990–2021	EAPC (95 % *CI*), 1990–2021	AAPC (95 % *CI*), 1990–2021
Global	1.05 (0.34**–**3.36)	0.07 (0.02**–**0.24)	−92.93 (−94.26, −90.65)	−6.84 (−7.78, −5.89)	−0.03 (−0.05, −0.02)
East Asia	0.07 (0.02**–**0.39)	0.03 (0.02**–**0.06)	−84.43 (−87.36, −80.45)	−5.44 (−5.75, −5.13)	−0.02 (−0.03, −0.01)
Southeast Asia	0.01 (0.00**–**0.01)	0.01 (0.01**–**0.02)	−83.40 (−85.96, −76.88)	−6.86 (−7.64, −6.07)	−0.02 (−0.02, −0.01)
Central Asia	0.03 (0.01**–**0.21)	0.04 (0.01**–**0.32)	52.03 (38.30**–**76.83)	1.73 (0.90**–**2.57)	0.02 (0.01**–**0.03)
Central Europe	0.12 (0.01**–**1.15)	0.01 (0.01**–**0.05)	−95.29 (−96.08, −94.36)	−10.93 (−11.84, −10.00)	−0.02 (−0.03, −0.01)
Western Europe	0.02 (0.01**–**0.15)	0.02 (0.01**–**0.04)	−71.68 (−74.91, −60.47)	−3.71 (−4.49, −2.92)	−0.02 (−0.03, −0.01)
Southern Latin America	0.01 (0.01**–**0.06)	0.01 (0.01**–**0.02)	−74.55 (−76.71, −72.75)	−3.29 (−4.18, −2.39)	−0.02 (−0.04, −0.01)
Caribbean	0.01 (0.01**–**0.02)	0.01 (0.01**–**0.02)	−90.81 (−92.91, −87.75)	−6.26 (−7.44, −5.06)	−0.02 (−0.03, −0.01)
Andean Latin America	0.18 (0.00**–**0.86)	0.02 (0.01**–**0.05)	−94.35 (−95.82, −92.57)	−7.39 (−8.52, −6.25)	−0.01 (−0.02, −0.01)
Central Latin America	0.02 (0.01**–**0.16)	0.02 (0.01**–**0.04)	−71.49 (−76.71, −62.45)	−5.37 (−6.32, −4.40)	−0.02 (−0.04, −0.01)
Tropical Latin America	0.66 (0.01**–**2.44)	0.51 (0.01**–**1.80)	−23.28 (−30.61, −4.30)	−1.45 (−1.78, −1.13)	−0.01 (−0.02, −0.01)
North Africa and Middle East	0.86 (0.01**–**6.88)	0.07 (0.02**–**0.56)	−92.36 (−94.51, −90.96)	−7.81 (−8.15, −7.46)	−0.03 (−0.03, −0.03)
South Asia	2.37 (0.01**–**10.32)	0.07 (0.01**–**0.35)	−97.06 (−98.03, −96.49)	−9.87 (−11.16, −8.56)	−0.07 (−0.08, −0.07)
Central Sub-Saharan Africa	5.51 (2.92**–**8.70)	0.37 (0.19**–**0.61)	−93.24 (−94.43, −91.86)	−2.85 (−5.59, −0.02)	−0.17 (−0.17, −0.16)
Eastern Sub-Saharan Africa	9.40 (6.40**–**12.93)	0.31 (0.19**–**0.47)	−96.66 (−97.51, −95.68)	−8.05 (−9.79, −6.27)	−0.31 (−0.31, −0.30)
Western Sub-Saharan Africa	0.01 (0.01**–**0.02)	0.04 (0.02**–**0.06)	312.44 (230.37**–**412.62)	7.28 (3.77**–**10.91)	0.01 (0.01**–**0.02)
High SDI	0.02 (0.01**–**0.06)	0.01 (0.01**–**0.03)	−93.01 (−99.26, −91.20)	−7.50 (−8.34, −6.66)	−0.01 (−0.02, −0.01)
High-middle SDI	0.05 (0.01**–**0.45)	0.02 (0.01**–**0.03)	−90.10 (−92.73, −50.61)	−7.13 (−7.72, −6.54)	−0.01 (−0.02, −0.01)
Middle SDI	0.13 (0.01**–**0.75)	0.05 (0.01**–**0.11)	−81.02 (−84.68, −42.02)	−5.23 (−5.53, −4.92)	−0.02 (−0.03, −0.01)
Low-middle SDI	0.96 (0.14**–**3.89)	0.09 (0.02**–**0.38)	−90.63 (−92.66, −88.63)	−6.82 (−7.44, −6.21)	−0.03 (−0.03, −0.01)
Low SDI	7.66 (2.79**–**21.15)	0.25 (0.10**–**0.59)	−96.80 (−97.64, −94.87)	−8.76 (−10.00, −7.50)	−0.25 (−0.26, −0.23)

Notes: The AAPC was ultimately utilized as the core statistical measure for evaluating trends in disease burden indicator. In the 21 GBD geographical regions, no data on the mortality rates for leishmaniasis have been reported from Eastern Europe, Australasia, high-income Asia Pacific, Southern Sub-Saharan Africa, Oceania, or high-income North America from 1990 to 2021. Abbreviations: AAPC, average annual percentage change; ASMR, age-standardized mortality rate; *CI*, confidence interval; EAPC, estimated annual percentage change; GBD, Global Burden of Disease; PC, percentage change; SDI, sociodemographic index; *UI*, uncertainty interval.

**Table 4:** The ASDR of leishmaniasis in 1990 and 2021 and its temporal trends across 21 GBD geographical regions.

Location	ASDR (per 100,000 population) (95 % *UI*), 1990	ASDR (per 100,000 population) (95 % *UI*), 2021	PC (95 % *UI*), 1990–2021	EAPC (95 % *CI*), 1990–2021	AAPC (95 % *CI*), 1990–2021
Global	79.59 (29.90**–**246.55)	10.27 (5.99**–**21.96)	−87.10 (−91.71, −76.67)	−5.17 (−6.03, −4.30)	−2.27 (−2.36, −2.17)
East Asia	5.20 (0.10**–**28.17)	0.82 (0.04**–**4.38)	−84.32 (−87.11, −49.48)	−5.48 (−5.76, −5.19)	−0.14 (−0.14, −0.14)
Southeast Asia	0.17 (0.04**–**0.52)	0.15 (0.09**–**0.22)	−12.87 (−64.11, 193.72)	−0.90 (−1.76, −0.03)	−0.02 (−0.03, −0.01)
Central Asia	15.47 (8.83**–**28.13)	9.43 (4.77**–**28.34)	−39.08 (−55.01, 0.65)	−1.76 (−1.90, −1.62)	−0.20 (−0.21, −0.19)
Central Europe	8.47 (0.07**–**80.58)	0.43 (0.03**–**3.79)	−94.88 (−95.75, −46.56)	−10.59 (−11.47, −9.70)	−0.26 (−0.26, −0.25)
Western Europe	1.57 (0.35**–**10.77)	0.49 (0.17**–**2.71)	−68.77 (−75.13, −48.54)	−3.69 (−4.45, −2.93)	−0.04 (−0.04, −0.03)
Southern Latin America	1.21 (0.30**–**4.68)	0.92 (0.51**–**1.82)	−24.03 (−60.58, 109.17)	−0.61 (−0.89, −0.34)	−0.01 (−0.02, −0.01)
High-income North America	0.01 (0.01**–**0.02)	0.01 (0.01**–**0.02)	10.89 (−41.49, 254.97)	0.44 (0.38**–**0.51)	−0.01 (−0.03, 0.01)
Caribbean	3.82 (1.93**–**6.95)	3.37 (2.19**–**4.92)	−11.63 (−38.39, 44.14)	−0.28 (−0.35, −0.22)	−0.01 (−0.02, −0.01)
Andean Latin America	47.21 (22.40**–**98.00)	35.47 (23.75**–**50.63)	−24.86 (−62.79, 22.66)	−0.66 (−0.76, −0.55)	−0.38 (−0.39, −0.37)
Central Latin America	16.86 (9.51**–**29.08)	14.12 (9.26**–**20.45)	−16.22 (−39.73, 13.79)	−0.48 (−0.56, −0.40)	−0.09 (−0.09, −0.08)
Tropical Latin America	64.36 (14.26**–**191.41)	52.47 (14.76**–**143.55)	−18.48 (−30.53, 14.26)	−1.07 (−1.32, −0.82)	−0.41 (−0.53, −0.29)
North Africa and Middle East	96.37 (27.75**–**519.24)	45.16 (28.39**–**79.84)	−53.14 (−84.56, 20.44)	−2.27 (−2.57, −1.98)	−1.76 (−1.80, −1.71)
South Asia	152.98 (1.40**–**693.40)	5.88 (0.85**–**26.13)	−96.16 (−97.13, −18.63)	−9.23 (−10.40, −8.04)	−4.75 (−4.97, −4.53)
Central Sub-Saharan Africa	389.40 (205.09**–**617.22)	26.79 (13.95**–**43.09)	−93.12 (−94.28, −91.77)	−2.87 (−5.55, −0.12)	−11.86 (−12.20, −11.52)
Eastern Sub-Saharan Africa	653.30 (442.46**–**909.04)	22.99 (13.88**–**34.59)	−96.48 (−97.36, −95.45)	−7.91 (−9.64, −6.13)	−20.49 (−21.20, −19.77)
Southern Sub-Saharan Africa	0.05 (0.02**–**0.11)	0.04 (0.03**–**0.06)	−10.43 (−44.78, 76.36)	−0.23 (−0.33, −0.12)	−0.01 (−0.02, −0.01)
Western Sub-Saharan Africa	2.94 (1.77**–**4.63)	5.54 (3.74**–**7.81)	88.35 (32.01**–**169.85)	2.53 (2.03**–**3.04)	0.08 (0.08**–**0.01)
High SDI	1.20 (0.33**–**4.89)	0.92 (0.55**–**1.43)	−23.52 (−75.18, 99.04)	−0.81 (−0.93, −0.69)	−0.02 (−0.01, −0.01)
High-middle SDI	4.34 (0.52**–**32.70)	1.10 (0.56**–**2.98)	−74.56 (−90.72, 24.95)	−4.16 (−4.73, −3.58)	−0.11 (−0.11, −0.06)
Middle SDI	14.00 (3.70**–**58.40)	8.33 (4.89**–**14.51)	−40.51 (−75.38, 47.74)	−1.55 (−1.73, −1.36)	−0.20 (−0.21, −0.19)
Low-middle SDI	73.17 (18.50**–**276.45)	12.24 (6.03**–**32.11)	−83.28 (−89.35, −61.05)	−5.18 (−5.62, −4.74)	−1.94 (−2.02, −1.87)
Low SDI	504.88 (200.59**–**1383.65)	25.20 (15.06**–**47.50)	−95.01 (−96.84, −91.30)	−7.53 (−8.78, −6.28)	−16.12 (−16.48, −15.75)

Notes: The AAPC was ultimately utilized as the core statistical measure for evaluating trends in disease burden indicator. In the 21 GBD geographical regions, no data on ASDR attributable to leishmaniasis have been reported in Eastern Europe, Australasia, Oceania, or high-income Asia Pacific from 1990 to 2021. Abbreviations: AAPC, average annual percentage change; ASDR, age-standardized disability-adjusted life years; *CI*, confidence interval; EAPC, estimated annual percentage change; GBD, Global Burden of Disease; PC, percentage change; SDI, sociodemographic index; *UI*, uncertainty interval.

### 3.2 Five SDI regions

In high SDI regions, both the ASIR and ASPR showed significant declines, with ASMR approaching zero and a reduction in ASDR. In high-middle SDI regions, the ASIR slightly increased, and the ASPR showed a modest rise, while both ASMR and ASDR decreased. In middle SDI regions, both the ASIR and ASPR increased, although ASMR, and the ASDR also saw a significant decline. In low-middle SDI regions, both the ASIR and ASMR decreased significantly, the ASPR slightly declined, and the ASDR experienced a substantial reduction. In low SDI regions, both the ASIR and ASPR decreased significantly, but ASMR remained high, with a considerable reduction in the ASDR (Table 1, Table 2, Table 3, Table 4).

From 1990 to 2021, in high SDI regions, the incidence, mortality, and DALYs have significantly decreased, reflecting effective disease control. In high-middle SDI regions, the incidence remains stable, while the mortality and DALYs have decreased. In middle SDI regions, both the incidence and prevalence increased, but mortality and DALYs decreased, indicating reduced disease burden. In low-middle SDI regions, incidence, mortality, and DALYs decreased substantially, signaling a clear reduction in disease burden. In low SDI regions, despite high mortality, incidence and prevalence decreased, and DALYs significantly declined, showing substantial overall improvement (Table S1–S4).

### 3.3 Geographical regions

From 1990 to 2021, the ASIR of leishmaniasis demonstrated significant geographical heterogeneity. Southeast Asia exhibited an upward trend in ASIR, while East Asia and Central Asia experienced a pronounced decline. In Latin America, multiple subregions—including the Andean, Central, and Tropical regions—also showed decreasing incidence rates. Similarly, North Africa, the Middle East, and Sub-Saharan Africa displayed an overall reduction in ASIR. Southern Latin America and high-income North America remained relatively stable with only marginal fluctuations, showing either consistent rates or slight decreases. The temporal patterns in incident case numbers mirrored these epidemiological trends: while the Andean and Central American regions reported substantial increases in case counts, Southeast Asia, Tropical Latin America, East Asia, Central Asia, and all three Sub-Saharan African regions (Western, Central, and Eastern) demonstrated significant declines in incident cases (Table 1, Table S1).

Southeast Asia and Western Sub-Saharan Africa exhibited an increasing trend in ASPR, while Southern Latin America and high-income North America demonstrated only marginal fluctuations. All other regions experienced a decline in ASPR. In addition, the number of prevalent cases exhibited distinct regional patterns: Eastern Sub-Saharan Africa, East Asia, Central Asia, Central Europe, and Western Europe all experienced declining case numbers, while all other regions demonstrated an upward trend (Table 2, Table S2).

Distinct mortality patterns emerged across regions: only Central Asia and Western Sub-Saharan Africa showed elevated ASMR and increasing death counts. Tropical Latin America maintained stable mortality numbers without significant variation, while all other regions experienced reductions in both ASMR and absolute death counts (Table 3, Table S3).

Our analysis has revealed substantial geographical disparities in ASDR trends (Tables 4 and S4). Western Sub-Saharan Africa emerged as the only region demonstrating a consistent increase in ASDR throughout the study period. In contrast, nine regions exhibited significant declines in ASDR, including: East Asia, Southeast Asia, Central Asia, Central Europe, Western Europe, North Africa and Middle East, South Asia, Central Sub-Saharan Africa, and Eastern Sub-Saharan Africa.

### 3.4 Countries and territories

From 1990 to 2021, This GBD 2021 study encompassing 101 endemic countries systematically evaluated the complete spectrum of leishmaniasis burden through four standardized metrics—ASIR, ASPR, ASMR, and ASDR—along with absolute counts of incident cases, prevalent cases, deaths, and DALY cases.

The study identified substantial geographical and temporal variations in the ASIR of leishmaniasis. In 2021, the Syrian Arab Republic exhibited the highest global ASIR (1967.12 per 100,000 population, 95 % *UI*: 1483.78**–**2534.28 per 100,000 population), followed by Afghanistan, Nicaragua, Tunisia, and Libya. Longitudinal assessment revealed divergent trends: 21 countries experienced significant ASIR increases, with Syria demonstrating the most pronounced rise (AAPC = 51.32 %, 95 % *CI*: 50.49 %**–**52.14 %), while 74 countries showed declining incidence, led by Somalia (AAPC = −10.71 %; 95 % *CI*: −10.87 %, −10.56 %). Six countries maintained stable ASIRs throughout the study period.

This study reveals significant global disparities in leishmaniasis burden, with the Syrian Arab Republic demonstrating the highest ASPR in 2021 at 4734.44 per 100,000 population (95 % *UI*: 4143.02**–**5554.19 per 100,000 population), substantially exceeding other high-burden countries including Afghanistan, Nicaragua, Panama, and Suriname. Longitudinal trends showed marked heterogeneity across countries, with 21 countries exhibiting significant ASPR increases (most notably Syria with an AAPC of 85.19 %, 95 % *CI*: 79.05 %**–**91.34 %), 74 countries showing declines (led by Turkmenistan at AAPC = −35.38 %; 95 % *CI*: −35.82 %, −34.95 %), and six maintaining stable rates.

Significant geographical disparities in leishmaniasis-related mortality are also revealed, with South Sudan demonstrating the highest ASMR globally in 2021 (6.13 per 100,000 population, 95 % *UI*: 3.18**–**9.92 per 100,000 population). The Central African Republic, Djibouti, and Niger emerged as subsequent high-burden nations. Longitudinal assessment identified divergent mortality patterns: 10 countries exhibited rising ASMRs, most notably Georgia with an AAPC of 0.01 % (95 % *CI*: 0.01 %**–**0.02 %), while 10 nations showed declining trends, led by the Central African Republic (AAPC = −0.35 %; 95 % *CI*: −0.36 %, −0.34 %). Notably, the majority of the monitored countries (*n* = 81) maintained stable mortality rates throughout the study period.

In 2021, South Sudan bore the highest ASDR (443.04 per 100,000 population, 95 % *UI*: 230.74**–**725.39 per 100,000 population), with Syria, the Central African Republic, Djibouti, and Nicaragua completing the top five highest-burden nations. Temporal analysis revealed divergent epidemiological trajectories: 10 countries experienced rising ASDRs, most markedly Syria with an AAPC of 4.78 % (95 % *CI*: 4.67 %**–**4.89 %), while 10 nations achieved significant reductions, led by South Sudan (AAPC = −140.82 %. 95 % *CI*: −157.95 %, −123.65 %). The majority of the monitored countries (*n* = 81) maintained stable disability burdens throughout the 31-year observation period (Table S5).

### 3.5 Global trends by age-gender group

In 2021, the total leishmaniasis exhibited marked age-dependent variations. Incidence and DALY rates followed a U-shaped trajectory, whereas prevalence rates increased steadily with age, and mortality rates displayed an L-shaped distribution. Among individuals under 70, no significant sex differences were observed in incidence or prevalence; however, in those aged 70 and older, both rates were higher in males. Although mortality rates were similar between sexes across all age groups, males under 85 experienced higher DALY rates (Fig. S1. A–D).

### 3.6 The association between different rates and SDI

For leishmaniasis, the ASIR, ASPR, ASMR, ASDR, and case counts were significantly negatively correlated with SDI (all *P* < 0.001) at both the 204-country level (2021) and the global plus 21-region level (1990–2021), with correlation coefficients ranging from approximately −0.26 to −0.56 (Table S6).

### 3.7 Projecting

Between 2022 and 2035, the global burden of leishmaniasis is projected to decline in terms of ASIR, ASMR, and ASDR (Table 5). In addition, leishmaniasis is projected to see an increase in the ASIR in several regions, including Southeast Asia, high-income North America, North Africa, and the Middle East, as well as in various parts of Sub-Saharan Africa (Eastern, Southern, and Western Sub-Saharan Africa) (Table S7).

**Table 5:** Projected burden of leishmaniasis based on BAPC model.

Index	Value (per 100,000 person-years) (95 % *CI*), 2035	EAPC (95 % *CI*), 2022–2035	AAPC (95 % *CI*), 2022–2035
ASIR	11.58 (6.16–16.99)	−1.13 (−1.15, −1.11)	−0.14 (−0.14, −0.12)
ASPR	85.78 (79.28–92.28)	0.86 (0.84–0.87)	0.69 (0.69–0.70)
ASMR	0.02 (0.01–0.04)	−8.75 (−8.76, −8.73)	−0.01 (−0.02, −0.01)
ASDR	3.24 (1.48–5.01)	−8.75 (−8.76, −8.73)	−0.455 (−0.46, −0.45)

Notes: The AAPC was ultimately utilized as the core statistical measure for evaluating trends in disease burden indicators. Abbreviations: AAPC: average annual percentage change; ASDR, age-standardized disability-adjusted life years; ASIR, age-standardized incidence rate; ASMR, age-standardized mortality rate; ASPR, age-standardized prevalence rate; BAPC: Bayesian age-period-cohort; *CI*: confidence intervals; EAPC: estimated annual percentage change; SDI, sociodemographic index.

### 3.8 Decomposition analysis

Globally, the DALYs burden of leishmaniasis decreased by 3,889,599.17, with epidemiological factors contributing to a 117.77 % reduction, reflecting the success of public health interventions. While population growth increased DALYs by 1,186,329.76, its impact was smaller (30.50 %) compared to epidemiological changes. Aging had a limited effect, accounting for 12.73 % of the overall change. Epidemiological interventions in low-SDI regions effectively reduced the DALYs burden, and similar positive effects were observed in middle-low SDI regions. In high-SDI regions, DALY changes were minimal, though population growth still affected the burden (Table 6).

**Table 6:** Decomposition analysis of the DALYs cases of leishmaniasis from 1990 to 2021.

Location	Overall difference	Aging	Aging (%)	population	Population (%)	Epidemiological change	Epidemiological change (%)
Global	−3,889,599.17	−495,071.67	12.73	1,186,329.76	−30.50	−4,580,857.26	117.77
Middle SDI	−42,459.08	−17,662.07	41.60	90,387.36	−212.88	−115,184.38	271.28
Low-middle SDI	−792,154.93	−26,550.81	3.35	276,729.18	−34.93	−1,042,333.30	131.58
High-middle SDI	−30,011.21	−9197.87	30.65	12,028.55	−40.08	−32,841.89	109.43
Low SDI	−3,024,596.98	799,314.04	−26.43	953,300.75	−31.52	−4,777,211.77	157.95
High SDI	849.35	−2217.52	−261.08	4037.35	475.35	−970.48	−114.26

Abbreviations: DALYS, disability-adjusted life-years; SDI, sociodemographic index.

### 3.9 Health inequality analysis

Leishmaniasis-related ASDR show significant absolute and relative inequalities across SDI levels. The gap in ASDR between the highest and lowest SDI regions decreased from −7.97 (95 % *CI*: −12.66, −3.28) in 1990 to −6.32 (95 % *CI*: −8.72, −3.92) in 2021, while the CCI improved from −0.73 (95 % *CI*: −0.82, −0.55) to −0.45 (95 % *CI*: −0.62, −0.24). Despite some progress, low-SDI countries continue to face a disproportionate disease burden, highlighting persistent global health inequalities.

### 3.10 Frontier analysis

**Fig. 1:**
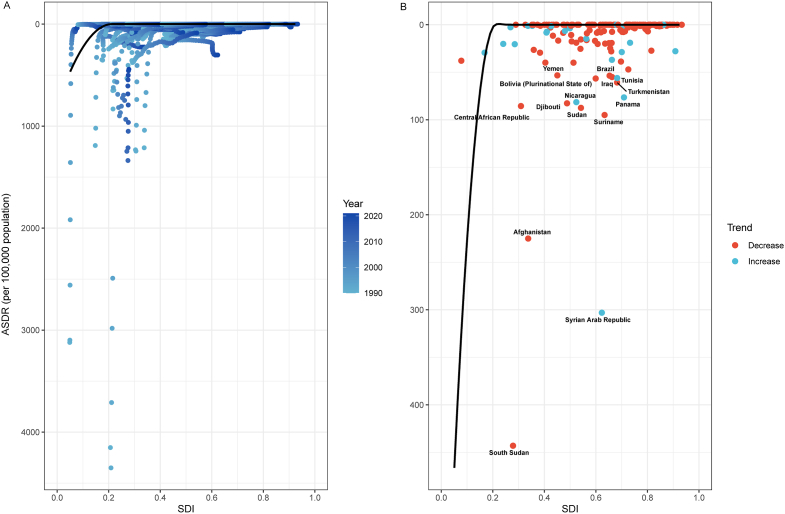
**Frontier analysis exploring the relationship between SDI and ASDR for leishmaniasis in 204 countries and territories**. It illustrates the relationship between SDI and ASDR for leishmaniasis from 1990 to 2021 across 204 countries and territories. A: The temporal trend showing the evolution of ASDR, with a color gradient representing different years: light blue for 1990 and dark blue for 2021. The gradient color reflects the changes in ASDR over time, with darker blue indicating the year 2021. B: This panel shows the association between ASDR and SDI for each country in 2021. Each point represents a specific country or territory, with a black frontier line indicating the optimal ASDR for the corresponding SDI. Blue points represent countries with a lower SDI, where ASDR has decreased from 1990 to 2021. Red points represent countries with a higher SDI, where ASDR has increased over time. Abbreviations: ASDR, age-standardized disability-adjusted life-years rate; SDI, sociodemographic index.

From 1990 to 2021, frontier analysis explored potential improvements in the ASDR of leishmaniasis across countries with varying SDI levels (Fig. 1). The several countries with the largest potential (effective different: ranging from 53.27 to 443.04) for improvement included South Sudan, Syrian Arab Republic, Afghanistan, Suriname, Sudan, Central African Republic, Djibouti, Nicaragua, Panama, Turkmenistan, Bolivia, Tunisia, Iraq, Brazil, and Yemen. High-SDI countries with notable improvement potential included Monaco and Saudi Arabia. While countries like Brazil and Panama showed a reduction in DALY rates, low-SDI countries, such as Afghanistan and South Sudan, experienced an increase in ASDR.

## 4 Discussion

This study presents a comprehensive analysis of global leishmaniasis trends. While age-standardized rates (ASIR, ASMR, and ASDR) show significant declines, incident and prevalent cases have risen substantially—a contrast to the marked reductions in mortality and DALY cases. These paradoxical trends suggest that improved case management and therapeutic interventions have effectively mitigated disease severity, yet persistent transmission challenges continue to hinder control of overall disease spread.

The increasing leishmaniasis burden in parts of North Africa, the Middle East, and Southeast Asia results from multiple interacting factors [22,23]. Firstly, prolonged conflict and political instability have severely damaged public health infrastructure in these regions, affecting disease prevention and treatment capabilities [24]. Large-scale population displacement has exacerbated disease transmission, especially in areas with poor sanitary conditions, such as refugee camps [25]. Furthermore, rapid urbanization and environmental degradation have created additional breeding grounds for sandflies, facilitating the spread of leishmaniasis [26]. The inadequate management of canine reservoirs, which are primary hosts for the disease, has further contributed to its spread [27]. Additionally, resource shortages and a lack of public health interventions present significant challenges for leishmaniasis control in these areas. Climate change has also played a catalytic role in expanding sandfly habitats, increasing the risk of outbreaks [27]. In summary, the rising burden of leishmaniasis highlights the urgent need for improvements in health governance, public health resource allocation, and environmental management in the region. Targeted interventions are urgently required to curb the spread of the disease.

The new strategy for leishmaniasis control, based on the One Health framework, should focus on a multi-dimensional and integrated management approach that takes into account the pathogen's characteristics and transmission patterns [28,29]. Vector control constitutes the pivotal component for interrupting the leishmaniasis transmission cycle, necessitating multi-modal intervention strategies. Environmental management focuses on eliminating sandfly breeding sites (e.g., organic waste, animal burrows, and structural crevices), while housing improvements (wall plastering and window screening) reduce indoor resting habitats. Chemical control employs pyrethroid compounds (e.g., deltamethrin) for residual spraying, complemented by the distribution of insecticide-treated nets and dog collars to minimize biting rates [30]. For biological control, entomopathogenic fungi such as *Beauveria bassiana* are applied for ecological regulation [31].

Canine reservoir host management requires an integrated approach combining diagnosis, treatment, and population control. Large-scale surveillance using rK39 rapid tests and PCR assays enables identification of infected dogs, with positive cases receiving antimonial compounds or miltefosine therapy [32]. In endemic areas, this may be supplemented with insecticide-impregnated collars. Vaccination, such as the Leish-Tec® vaccine approved in Brazil, serves as a complementary intervention [33]. Wildlife surveillance should focus on potential reservoir species including foxes and rodents, employing serological surveys and spatial analysis to elucidate their epidemiological significance [34]. Regarding ethical considerations, the benefit-risk ratio of canine culling strategies requires careful evaluation based on local transmission dynamics.

The diagnostic system for early-stage leishmaniasis should be decentralized to primary healthcare levels, with widespread implementation of rK39 rapid tests and loop-mediated isothermal amplification (Loopamp) technologies. To ensure therapeutic accessibility, prioritized supply chains must be established for liposomal amphotericin B and miltefosine. Community engagement mechanisms should focus on training frontline health workers to recognize hallmark clinical manifestations (e.g., prolonged fever and splenomegaly), while concurrently strengthening public health education to enhance protective awareness [2,35].

The control strategy should be tailored to regional transmission patterns, as areas with rapid urbanization and significant ecological degradation require specific interventions. In such areas, improving environmental sanitation and waste management is vital. In conflict-prone regions, it is necessary to strengthen surveillance and intervention efforts, while also providing adequate medical support. In addition, for high-risk displaced populations, enhanced health protection and medical assistance are crucial. The One Health framework also emphasizes the need to improve public health infrastructure, particularly in low-income and resource-limited areas, where public health education and disease prevention awareness must be prioritized alongside basic medical services [2,36].

Advancements in technology have introduced innovative tools for disease surveillance, transmission prediction, and resource allocation, which can greatly enhance leishmaniasis control [37]. The application of big data, artificial intelligence, and remote sensing technologies allows for precise monitoring of vectors and forecasting of transmission trends [38]. Furthermore, promoting international data sharing and collaboration will help establish a global control network, offering essential data support for the development of efficient and targeted prevention strategies.

Overall, the One Health-based approach to leishmaniasis control emphasizes interdisciplinary collaboration, integrating vector control, reservoir management, environmental sanitation, and human health interventions to create a more systematic and sustainable framework for disease control [39].

This study has several notable limitations. First, the accuracy of disease burden estimates within the GBD 2021 framework is highly dependent on the quality and availability of input data. In particular, low- and middle-income countries often face resource constraints, leading to underreporting or misreporting of leishmaniasis, which may introduce systematic biases and affect the accuracy of global and regional estimates [11,12]. Second, the GBD estimates rely on long-term equilibrium assumptions and static parameter settings. In cases of insufficient data, the model extrapolates trends from geographically adjacent regions, which may result in estimates that do not accurately reflect actual disease burden [[11], [12], [13]]. Third, GBD 2021 tends to overestimate the disease burden [40,41]. Future research should prioritize standardizing data collection and analytical methods for leishmaniasis, particularly in countries and regions with significant disparities in development and surveillance capabilities. A disease burden framework tailored to the specific conditions of each country or region is necessary to accurately assess the disease burden across different settings.

## 5 Conclusion

This study offers a comprehensive assessment of the global burden of leishmaniasis, underscoring persistent regional disparities in disease distribution. The findings reveal that leishmaniasis remains a major public health threat, particularly in South Asia. Addressing this challenge requires strengthening public health interventions, optimizing resource allocation, and improving health governance in low- and middle-income countries. Moreover, implementing an integrated One Health approach—emphasizing vector control, reservoir host management, and environmental sanitation—is critical to reducing disease transmission and achieving sustainable control.

## CRediT authorship contribution statement

**Shunxian Zhang:** Writing – review & editing, Writing – original draft, Methodology. **Guobing Yang:** Writing – review & editing, Writing – original draft. **Shan Lv:** Writing – review & editing, Writing – original draft, Formal analysis. **Lei Duan:** Visualization, Validation, Supervision, Data curation. **Muxin Chen:** Data curation. **Qin Liu:** Investigation. **Liguang Tian:** Data curation. **Shizhu Li:** Project administration, Conceptualization. **Jinxin Zheng:** Writing – review & editing, Writing – original draft, Methodology, Formal analysis.

## Ethics approval and consent to participate

This study only used publicly available, de-identified national-level aggregated data and did not involve any human subjects, nor were any interventional experiments or individual-level data collection conducted. According to the evaluation by the Ethics Review Committee of our institution, this study was classified as not involving human subjects and therefore exempt from ethical review.

## Data availability statement

The datasets analyzed during the current study are available at http://ghdx.healthdata.org/gbd-results-tool.

## Consent for publication

All authors consent for publication.

## Funding sources

The study was supported by the Three-Year Initiative Plan for Strengthening Public Health System Construction in Shanghai (2023–2025) Key Discipline Project (grant number GWVI-11.1-12), the International Joint Laboratory on Tropical Diseases Control in Greater Mekong Subregion from Shanghai Municipality Government (grant number 21410750200), the Traditional Chinese Medicine Innovation Team of Shanghai Municipal Health Commission (grant number 2022CX010), Talent Fund of Longhua Hospital affiliated to Shanghai University of Traditional Chinese Medicine (grant number LH001.007), Shanghai Natural Science Foundation (grant number 23ZR1464000), Science and technology development project of Shanghai University of traditional Chinese medicine (grant number 24BZH07), Excellent Academic Leaders Program of Shanghai Science and Technology Commission (grant number 22XD1423500), Multidisciplinary Innovation Team of Traditional Chinese Medicine of China (grant number ZYYCXTD-D-202208), the 2023 Xuhui District Project (grant number 23XHYD-25). The Funders had no role in the study design or in the collection, analysis, and interpretation of the data, writing of the report, or decision to submit the article for publication.

## Declaration of competing interest

The authors have declared that no competing interests exist.
