# Supplementary material for: Global, regional, and national temporal trend and patterns of change in the burden of leishmaniasis from 1990 to 2021: an analysis of the Global Burden of Disease Study 2021
Source: Sci One Health. 2025 Sep 6;4:100123. doi: 10.1016/j.soh.2025.100123 (PMC12482278; doi:10.1016/j.soh.2025.100123)
Supplement: Multimedia component 1 [file mmc1.docx]

**Supporting Information**

**Global, regional, and national temporal trend and patterns of change in the burden of leishmaniasis from 1990 to 2021: an analysis of the Global Burden of Disease Study 2021**

Shunxian Zhang^a,1,^ Guobing Yang^b,1^, Shan Lv^c,1^, Lei Duan^c^, Muxin Chen^c^, Qin Liu^c^, Liguang Tian^c^, Shizhu Li^c,^*, and Jinxin Zheng^c,**^

^a^ Longhua Hospital, Shanghai University of Traditional Chinese Medicine, Shanghai 200032, China

^b^ Gansu Provincial Center for Disease Control and Prevention, Gansu Provincial Academy of Preventive Medicine, Lanzhou 730000, Gansu, China

^C^ National Key Laboratory of Intelligent Tracking and Forecasting for Infectious Diseases, NHC Key Laboratory of Parasite and Vector Biology, WHO Collaborating Centre for Tropical Diseases, National Center for International Research on Tropical Diseases, National Institute of Parasitic Diseases of Chinese Center for Disease Control and Prevention, Shanghai 200025, China

^*^Corresponding author.

^**^Corresponding author.

*Email addresses:* [zhangshunxian110@163.com](mailto:zhangshunxian110@163.com) (S. Zhang), [gostly_1@163.com (G](mailto:gostly_1@163.com%20(G). Yang), lvshan@nipd.chinacdc.cn (S. Lv), duanlei@nipd.chinacdc.cn (L. Duan), [liuqin@nipd.chinacdc.cn](mailto:liuqin@nipd.chinacdc.cn) (Q. Liu), [chenmx@chinacdc.cn](mailto:chenmx@chinacdc.cn) (M. Chen), [tianlg@nipd.chinacdc.cn](mailto:tianlg@nipd.chinacdc.cn) (L. Tian), [lisz@chinacdc.cn](mailto:lisz@chinacdc.cn) (S. Li), [jamesjin63@163.com](mailto:jamesjin63@163.com) (J. Zheng)

^1^ Shunxian Zhang, Guobing Yang and Shan Lv contributed equally to this work.

**Items**

Table S1. The incident cases of Leishmaniasis in 2021, and the changing trends of incidence cases were analyzed across GBD regions.

Table S2. The prevalent cases of leishmaniasis in 1990 and 2021, and the changing trends of prevalent cases were analyzed across 21 GBD geographical regions.

Table S3. The mortality cases of leishmaniasis in 1990 and 2021, and change trend of death cases were analyzed across 21 GBD geographical regions.

Table S4. The DALYs case of leishmaniasis in 1990 and 2021, and change trend of DALYs cases were analyzed across 21 GBD geographical regions.

Table S5. ASRs and case counts for leishmaniasis, and the AAPC of ASRs and case counts for 204 countries and territories (highest five and lowest five).

Fig S1. The specific rate of leishmaniasis showed notable differences across age and gender distributions in 2021 year (A: incidence rate, B: prevalence, C: mortality rate, D: DALY rate). Abbreviations: DALYs, disability-adjusted life years; *UI*, uncertainty interval.

Table S6. The association between the burden of leishmaniasis and the SDI in global.

Table S7. Based on the BAPC model, the trends in the disease burden indicators for leishmaniasis from 2022 to 2035 were predicted in global and 21 GBD geographical regions.

**Table S1.** The incident cases of Leishmaniasis in 2021, and the changing trends of incidence cases were analyzed across GBD regions.

| Location | Incident cases (*n*) (95% UI), 1990 | Incident cases (*n*) (95% *UI*), 2021 | PC (95% *CI*), 1990–2021 | EAPC (95% *CI*), 1990–2021. | AAPC (95% *CI*), 1990–2021 |
| --- | --- | --- | --- | --- | --- |
| Global | 1011925(859813,1190054) | 1096860(969151,1243291) | 8.39(-10.55,31.42) | 0.23(-0.38,0.85) | 2103.21(911.89,3294.53) |
| East Asia | 9546(6509,15142) | 1952(1236,3276) | -79.55(-89.27,-59.62) | -4.81(-5.76,-3.84) | -243.74(-248.06,-239.42) |
| Southeast Asia | 821(437,1433) | 7928(6079,10012) | 865.71(425.66,1664.86) | 7.59(5.81,9.40) | 229.55(222.81,236.29) |
| Central Asia | 21030(16098,27940) | 6801(5102,9102) | -67.66(-78.09,-52.08) | -2.86(-4.45,-1.24) | -453.89(-482.63,-425.16) |
| Central Europe | 1854(1121,3521) | 156(118,202) | -91.58(-95.58,-84.66) | -8.52(-9.12,-7.92) | -54.63(-55.53,-53.74) |
| Western Europe | 1490(1157,1988) | 1171(967,1468) | -21.36(-45.28,12.50) | -0.84(-1.10,-0.57) | -9.55(-10.58,-8.52) |
| Southern Latin America | 963(399,1951) | 1185(719,1736) | 23.00(-48.73,244.41) | 0.08(-0.43,0.60) | 11.38(8.07,14.70) |
| High-income North America | 66(15,170) | 112(25,320) | 70.90(-64.90,905.02) | 2.07(1.62,2.52) | 1.62(1.55,1.69) |
| Caribbean | 2613(1406,4661) | 1899(1119,3292) | -27.33(-64.04,72.60) | -0.84(-1.34,-0.33) | -30.56(-43.31,-17.82) |
| Andean Latin America | 30913(22121,42993) | 40478(32343,50673) | 30.94(-9.52,95.09) | -1.75(-2.09,-1.42) | 302.72(273.76,331.67) |
| Central Latin America | 61806(43816,89087) | 67882(54663,85933) | 9.83(-25.42,67.65) | 0.33(-0.70,1.37) | 386.01(167.43,604.59) |
| Tropical Latin America | 69422(55212,88128) | 66220(56144,77742) | -4.61(-29.68,29.02) | -1.41(-1.98,-0.85) | -113.88(-183.47,-44.30) |
| North Africa and Middle East | 362362(277402,482485) | 795885(679693,926269) | 119.64(58.72,202.04) | 1.68(1.11,2.25) | 14245.68(13758.67,14732.69) |
| South Asia | 284845(199926,387743) | 66162(41725,101317) | -76.77(-86.30,-59.28) | -5.38(-6.25,-4.51) | -6693.48(-7103.16,-6283.80) |
| Central Sub-Saharan Africa | 25243(9029,62385) | 5586(2747,11215) | -77.87(-93.06,-33.28) | -3.32(-5.37,-1.23) | -631.46(-647.99,-614.93) |
| Eastern Sub-Saharan Africa | 131180(80729,203562) | 15424(12750,18706) | -88.24(-92.94,-80.28) | -6.84(-8.33,-5.33) | -3721.98(-3798.45,-3645.52) |
| Southern Sub-Saharan Africa | 56(24,108) | 68(31,134) | 20.48(-53.33,307.71) | -0.08(-0.56,0.39) | 0.38(0.28,0.48) |
| Western Sub-Saharan Africa | 7716(4788,11922) | 17951(13533,23936) | 132.64(43.64,287.44) | -0.83(-1.38,-0.28) | 324.18(305.85,342.50) |
| High SDI | 22141(9878,43119) | 5527(2868,10258) | -75.04(-89.53,-33.25) | -4.67(-5.50,-3.84) | -541.75(-609.00,-474.49) |
| High-middle SDI | 28512(18393,45234) | 25655(16409,38660) | -10.02(-52.05,62.06) | -0.17(-1.19,0.87) | -0.83(-66.91,65.25) |
| Middle SDI | 269020(204094,381665) | 572243(494087,663068) | 112.71(51.02,192.10) | 2.00(1.52,2.47) | 9998.93(9649.13,10348.73) |
| Low-middle SDI | 305212(244572,392078) | 193110(158191,236871) | -36.73(-54.27,-15.42) | -2.61(-3.03,-2.18) | -3504.91(-3702.94,-3306.88) |
| Low SDI | 386626(292249,501146) | 299491(220529,399042) | -22.54(-48.32,18.10) | -0.93(-2.22,0.38) | -3118.29(-3835.92,-2400.66) |

Notes: In 21 GBD geographical regions, from 1990 to 2021, no data on the incidence cases of leishmaniasis have been reported from Oceania, Eastern Europe, the high-income Asia Pacific, or Australasia. Abbreviations: AAPC, average annual percent change; *CI*, confidence interval; GBD, Global Burden of Disease; UI, uncertainty interval; SDI, sociodemographic index.

**Table S2.** The prevalent cases of leishmaniasis in 1990 and 2021, and the changing trends of prevalent cases were analyzed across 21 GBD geographical regions.

| Location | Prevalent cases (*n*) (95% *UI*), 1990 | Prevalent cases (*n*) (95% *UI*), 2021 | PC (95% *CI*), 1990–2021 | EAPC (95% *CI*), 1990–2021 | AAPC (95% *CI*), 1990–2021 |
| --- | --- | --- | --- | --- | --- |
| Global | 3184236(2658032,3883766) | 6209396(5825066,6694051) | 95.00(69.01,121.80) | 0.96(0.90,1.01) | 97225.09(96608.04,97842.14) |
| East Asia | 27417(12030,65837) | 20540(10592,44226) | -25.08(-34.95,-2.64) | -1.72(-1.80,-1.64) | -223.60(-227.60,-219.60) |
| Southeast Asia | 4065(2036,7308) | 14510(12370,16710) | 256.99(116.33,560.12) | 3.13(2.48,3.79) | 335.79(331.06,340.53) |
| Central Asia | 135942(103471,180370) | 103350(86278,124641) | -23.97(-31.42,-13.26) | -2.28(-2.39,-2.18) | -1081.45(-1112.33,-1050.58) |
| Central Europe | 1761(1232,2557) | 1021(841,1295) | -41.98(-52.51,-26.88) | -1.58(-1.65,-1.52) | -24.50(-24.85,-24.15) |
| Western Europe | 4243(2536,7162) | 4139(3224,5622) | -2.45(-21.14,29.37) | -0.57(-0.63,-0.51) | -3.16(-3.87,-2.45) |
| Southern Latin America | 6265(2480,13787) | 9481(7455,13117) | 51.33(-5.30,208.47) | 0.37(0.13,0.62) | 103.93(100.07,107.78) |
| High-income North America | 266(62,695) | 412(254,692) | 54.81(-9.25,339.35) | 0.62(0.55,0.68) | 4.76(4.70,4.82) |
| Caribbean | 18121(9853,31602) | 26183(21334,33101) | 44.49(4.46,127.29) | 0.25(0.20,0.30) | 261.24(255.47,267.02) |
| Andean Latin America | 172242(121747,246948) | 357494(317878,403132) | 107.55(59.62,172.59) | 0.45(0.26,0.64) | 5973.83(5944.44,6003.22) |
| Central Latin America | 321446(228287,458657) | 552350(494121,640116) | 71.83(36.26,120.46) | 0.54(0.41,0.67) | 7396.84(7284.32,7509.35) |
| Tropical Latin America | 409734(312997,529907) | 736075(667662,817840) | 79.65(50.85,116.85) | 0.58(0.40,0.76) | 10557.92(10347.10,10768.73) |
| North Africa and Middle East | 1741351(1285783,2366455) | 3831680(3531870,4202149) | 120.04(73.69,184.05) | 0.63(0.54,0.73) | 66639.55(65756.58,67522.53) |
| South Asia | 227062(151552,324909) | 342974(292952,406609) | 51.05(15.07,107.92) | -0.29(-0.55,-0.03) | 3877.03(3730.76,4023.30) |
| Central Sub-Saharan Africa | 13597(6532,25789) | 17413(13746,22289) | 28.06(-26.28,147.84) | -1.04(-1.48,-0.59) | 122.26(116.59,127.93) |
| Eastern Sub-Saharan Africa | 49992(33224,71156) | 36553(31756,42208) | -26.88(-47.35,1.83) | -2.58(-3.20,-1.96) | -401.96(-426.98,-376.94) |
| Southern Sub-Saharan Africa | 310(133,602) | 498(390,640) | 60.71(0.92,223.44) | 0.26(0.18,0.35) | 6.08(5.96,6.19) |
| Western Sub-Saharan Africa | 50424(31213,77806) | 154722(137890,173271) | 206.84(114.73,370.35) | 0.69(0.35,1.03) | 3375.23(3333.81,3416.65) |
| High SDI | 104688(44757,208183) | 165817(119646,236689) | 58.39(11.04,190.98) | 0.60(0.50,0.69) | 1970.23(1936.44,2004.02) |
| High-middle SDI | 127128(73541,224190) | 172417(139434,227241) | 35.62(-0.17,99.26) | 0.31(0.22,0.40) | 1393.71(1332.15,1455.27) |
| Middle SDI | 1273349(955701,1762633) | 2691876(2506745,2975572) | 111.40(66.83,163.52) | 1.35(1.24,1.46) | 44448.84(43691.80,45205.89) |
| Low-middle SDI | 1285311(964357,1851904) | 1756325(1567187,2023530) | 36.65(10.68,65.55) | -0.81(-0.88,-0.73) | 15055.20(14765.95,15344.45) |
| Low SDI | 392119(276495,573570) | 1418428(1282334,1567000) | 261.73(158.97,399.32) | 2.44(2.03,2.86) | 33778.54(33301.72,34255.37) |

Notes: In 21 GBD geographical regions, from 1990 to 2021, no data on the number of prevalent cases of leishmaniasis have been reported from Oceania, Eastern Europe, the high-income Asia Pacific, or Australasia. Abbreviations: AAPC, average annual percent change; *CI*, confidence interval; GBD, Global Burden of Disease; *UI*, uncertainty interval; SDI, sociodemographic index.

**Table S3.** The mortality cases of leishmaniasis in 1990 and 2021, and change trend of death cases were analyzed across 21 GBD geographical regions.

| Location | Deaths (*n*) (95% UI), 1990 | Deaths (*n*) (95% UI), 2021 | PC (95% *CI*), 1990–2021 | EAPC (95% *CI*). 1990–2021 | AAPC (95% *CI*), 1990–2021 |
| --- | --- | --- | --- | --- | --- |
| Global | 60245(20096,193319) | 5482(1609,17781) | -90.90(-92.73,-88.50) | -7.25(-8.19,-6.30) | -1803.48(-1885.59,-1721.37) |
| East Asia | 864(0,4752) | 144(0,759) | -83.31(-85.84,-74.59) | -5.83(-6.20,-5.47) | -23.33(-23.76,-22.89) |
| Southeast Asia | 8(0,34) | 2(0,7) | -76.61(-81.28,-60.57) | -7.05(-7.84,-6.26) | -0.19(-0.20,-0.18) |
| Central Asia | 18(0,163) | 36(0,308) | 98.04(85.94,148.37) | 1.55(0.86,2.25) | 0.60(0.54,0.66) |
| Central Europe | 145(0,1310) | 6(0,49) | -95.96(-96.34,-94.59) | -11.17(-12.04,-10.28) | -4.41(-4.49,-4.33) |
| Western Europe | 83(21,495) | 26(7,142) | -68.88(-75.05,-59.83) | -3.51(-4.30,-2.71) | -1.97(-2.24,-1.69) |
| Southern Latin America | 3(0,29) | 1(0,9) | -69.27(-70.44,-64.94) | -3.65(-4.52,-2.77) | -0.07(-0.08,-0.07) |
| Caribbean | 2(0,8) | 0(0,1) | -88.80(-90.80,-83.08) | -6.61(-7.79,-5.41) | -0.05(-0.05,-0.05) |
| Andean Latin America | 76(0,390) | 6(0,31) | -91.56(-93.57,-87.73) | -7.90(-9.03,-6.76) | -2.23(-2.27,-2.20) |
| Central Latin America | 45(0,310) | 17(0,105) | -63.07(-68.89,-44.73) | -5.88(-6.83,-4.92) | -0.88(-0.98,-0.78) |
| Tropical Latin America | 1072(0,4133) | 1077(0,3748) | 0.46(-11.73,42.24) | -1.89(-2.20,-1.58) | 2.17(-2.06,6.41) |
| North Africa and Middle East | 3223(0,27202) | 411(0,3547) | -87.26(-90.27,-84.15) | -8.02(-8.37,-7.66) | -97.82(-100.99,-94.66) |
| South Asia | 27848(5,126974) | 1252(0,6490) | -95.50(-96.93,-94.63) | -10.20(-11.48,-8.90) | -865.63(-911.11,-820.15) |
| Central Sub-Saharan Africa | 3904(2040,6207) | 613(312,998) | -84.30(-86.97,-81.14) | -2.99(-5.73,-0.17) | -109.29(-113.23,-105.35) |
| Eastern Sub-Saharan Africa | 22930(15416,32154) | 1639(969,2492) | -92.85(-94.74,-90.68) | -8.13(-9.86,-6.37) | -731.08(-757.80,-704.37) |
| Western Sub-Saharan Africa | 25(13,40) | 253(132,418) | 930.24(728.19,1192.40) | 7.33(3.82,10.96) | 7.35(7.11,7.59) |
| High SDI | 55(6,414) | 5(0,35) | -91.56(-99.30,-88.54) | -6.93(-8.13,-5.71) | -1.72(-2.05,-1.38) |
| High-middle SDI | 537(13,4613) | 60(7,354) | -88.90(-92.30,-45.92) | -7.36(-7.99,-6.72) | -16.51(-17.34,-15.68) |
| Middle SDI | 2346(1,14066) | 574(0,2620) | -75.55(-81.50,-28.20) | -5.54(-5.83,-5.25) | -61.75(-64.11,-59.38) |
| Low-middle SDI | 12690(2073,52418) | 1729(242,7482) | -86.38(-90.04,-83.22) | -7.20(-7.80,-6.59) | -352.34(-367.50,-337.17) |
| Low SDI | 44596(17669,122184) | 3114(1306,7359) | -93.02(-95.01,-88.89) | -8.76(-10.00,-7.50) | -1411.55(-1449.85,-1373.25) |

Notes: In 21 GBD geographical regions, from 1990 to 2021, no data on the mortality cases for leishmaniasis have been reported from Eastern Europe, Australasia, high-income Asia Pacific, Southern Sub-Saharan Africa, Oceania, or High-income North America. Abbreviations: *CI*, confidence interval; GBD, Global Burden of Disease; *UI*, uncertainty interval; SDI, sociodemographic index.

**Table S4.** The DALYs case of leishmaniasis in 1990 and 2021, and change trend of DALYs cases were analyzed across 21 GBD geographical regions.

| Location | DALYs cases (*n*) (95% *UI*), 1990 | DALYs cases (*n*) (95% *UI*), 2021 | PC (95% *CI*), 1990–2021 | EAPC (95% *CI*), 1990–2021 | AAPC (95% *CI*), 1990–2021 |
| --- | --- | --- | --- | --- | --- |
| Global | 4670787(1752403,14551294) | 781188(468168,1636592) | -83.28(-89.49,-68.91) | -5.56(-6.45,-4.66) | -128206.44(-134253.11,-122159.77) |
| East Asia | 63324(1122,347568) | 9822(762,48836) | -84.49(-87.60,-20.60) | -6.14(-6.48,-5.80) | -1737.00(-1767.08,-1706.91) |
| Southeast Asia | 790(147,2755) | 1028(626,1518) | 30.17(-52.81,435.55) | -0.94(-1.90,0.03) | 10.05(8.80,11.31) |
| Central Asia | 9951(5549,19983) | 8871(4428,27279) | -10.85(-32.71,38.00) | -1.57(-1.69,-1.45) | -44.26(-51.34,-37.17) |
| Central Europe | 9269(94,87956) | 386(48,2980) | -95.84(-96.73,-38.21) | -10.92(-11.72,-10.11) | -282.32(-287.26,-277.38) |
| Western Europe | 4808(1135,31830) | 1646(593,8296) | -65.77(-74.22,-42.76) | -3.71(-4.41,-2.99) | -104.35(-117.23,-91.48) |
| Southern Latin America | 593(144,2370) | 657(368,1169) | 10.68(-50.59,211.77) | -0.42(-0.66,-0.17) | 1.16(0.50,1.82) |
| High-income North America | 18(4,47) | 28(14,50) | 55.02(-9.25,339.35) | 0.62(0.56,0.69) | 0.32(0.32,0.32) |
| Caribbean | 1275(637,2317) | 1661(1079,2444) | 30.35(-11.02,109.68) | 0.04(-0.03,0.11) | 12.68(12.24,13.12) |
| Andean Latin America | 16772(7222,40696) | 23162(15488,32893) | 38.10(-42.37,154.51) | -0.33(-0.47,-0.19) | 208.22(203.90,212.53) |
| Central Latin America | 23844(12790,46237) | 36035(23656,52083) | 51.13(-6.24,114.96) | 0.05(-0.03,0.13) | 388.88(378.64,399.12) |
| Tropical Latin America | 103878(19521,330331) | 114003(36297,294025) | 9.75(-15.45,103.26) | -1.42(-1.65,-1.19) | 276.59(36.59,516.59) |
| North Africa and Middle East | 346970(76374,2169156) | 272526(170484,492504) | -21.46(-77.11,165.46) | -2.51(-2.89,-2.13) | -2957.37(-3193.99,-2720.75) |
| South Asia | 2002800(13705,9288452) | 106312(15611,478610) | -94.69(-96.09,48.31) | -9.84(-11.01,-8.65) | -61743.35(-64913.93,-58572.77) |
| Central Sub-Saharan Africa | 303775(159648,485337) | 47360(24257,77017) | -84.41(-86.96,-81.34) | -3.04(-5.75,-0.27) | -8512.18(-8815.98,-8208.39) |
| Eastern Sub-Saharan Africa | 1777540(1188355,2524593) | 127773(76095,194043) | -92.81(-94.72,-90.59) | -8.11(-9.83,-6.36) | -56622.17(-58683.13,-54561.20) |
| Southern Sub-Saharan Africa | 20(7,45) | 32(19,47) | 59.04(-5.06,222.65) | 0.25(0.16,0.34) | 0.38(0.37,0.39) |
| Western Sub-Saharan Africa | 5160(3221,7959) | 29889(19044,44057) | 479.29(307.03,680.98) | 3.45(2.62,4.28) | 814.18(790.48,837.87) |
| High SDI | 9866(2861,35756) | 10715(6453,17389) | 8.61(-63.09,153.76) | -0.39(-0.52,-0.26) | 26.98(19.32,34.65) |
| High-middle SDI | 44319(5500,332670) | 14308(7590,31873) | -67.72(-90.13,60.01) | -4.00(-4.64,-3.35) | -1047.68(-1106.92,-988.43) |
| Middle SDI | 247701(56758,1119519) | 205242(122915,343125) | -17.14(-69.75,144.23) | -1.60(-1.82,-1.37) | -1721.38(-1930.11,-1512.66) |
| Low-middle SDI | 1021708(237344,4006642) | 229553(110913,630090) | -77.53(-85.57,-44.94) | -5.80(-6.26,-5.33) | -25469.56(-26569.50,-24369.63) |
| Low SDI | 3345537(1379709,9050697) | 320940(183604,622157) | -90.41(-93.70,-83.98) | -7.90(-9.15,-6.63) | -102758.10(-105444.36,-100071.83) |

Notes: In 21 GBD geographical regions, from 1990 to 2021, no data on DALY cases attributable to leishmaniasis have been reported from Eastern Europe, Australasia, Oceania, or the high-income Asia Pacific. Abbreviations: DALYs, disability-adjusted life years; *CI*, confidence interval; GBD, Global Burden of Disease; *UI*, uncertainty interval; SDI, sociodemographic index.

**Table S5.** ASRs and case counts for leishmaniasis, and the AAPC of ASRs and case counts for 204 countries and territories (highest five and lowest five).

| Feature | Rank | Year | Index | Rate | Nations | Value (95% UI) |
| --- | --- | --- | --- | --- | --- | --- |
| ascending | 1 | 1990-2021 | ASIR | Rate | Djibouti | 213.128(115.614,310.643) |
| ascending | 2 | 1990-2021 | ASIR | Rate | Zimbabwe | 110.566(4.625,216.507) |
| descending | 1 | 1990-2021 | ASIR | Rate | Bhutan | -2477.506(-2549.230,-2405.782) |
| descending | 2 | 1990-2021 | ASIR | Rate | Republic of Vanuatu | -1081.616(-1231.572,-931.660) |
| descending | 3 | 1990-2021 | ASIR | Rate | Papua New Guinea | -812.335(-915.163,-709.507) |
| descending | 4 | 1990-2021 | ASIR | Rate | Guinea-Bissau | -744.008(-796.740,-691.276) |
| descending | 5 | 1990-2021 | ASIR | Rate | Ghana | -738.946(-794.955,-682.936) |
| ascending | 1 | 1990-2021 | ASPR | Rate | Djibouti | 84.486(72.460,96.511) |
| ascending | 2 | 1990-2021 | ASPR | Rate | Ethiopia | 17.170(0.766,33.573) |
| ascending | 3 | 1990-2021 | ASPR | Rate | Zimbabwe | 15.632(-8.844,40.108) |
| ascending | 4 | 1990-2021 | ASPR | Rate | Bolivarian Republic of Venezuela | 13.471(10.548,16.394) |
| ascending | 5 | 1990-2021 | ASPR | Rate | Korea | 10.901(4.812,16.989) |
| descending | 1 | 1990-2021 | ASPR | Rate | Burkina Faso | -1166.759(-1291.799,-1041.719) |
| descending | 2 | 1990-2021 | ASPR | Rate | Ghana | -1120.287(-1178.161,-1062.412) |
| descending | 3 | 1990-2021 | ASPR | Rate | C么te d'Ivoire | -829.183(-850.466,-807.900) |
| descending | 4 | 1990-2021 | ASPR | Rate | Guinea-Bissau | -791.863(-824.868,-758.858) |
| descending | 5 | 1990-2021 | ASPR | Rate | Bhutan | -747.430(-758.320,-736.540) |
| ascending | 1 | 1990-2021 | ASMR | Rate | Central African Republic | 1.374(1.251,1.497) |
| ascending | 2 | 1990-2021 | ASMR | Rate | Benin | 1.309(1.119,1.499) |
| ascending | 3 | 1990-2021 | ASMR | Rate | Angola | 0.382(0.233,0.530) |
| ascending | 4 | 1990-2021 | ASMR | Rate | South Sudan | 0.346(0.218,0.473) |
| ascending | 5 | 1990-2021 | ASMR | Rate | Republic of Nicaragua | 0.034(0.029,0.039) |
| descending | 1 | 1990-2021 | ASMR | Rate | Malawi | -3.981(-4.302,-3.660) |
| descending | 2 | 1990-2021 | ASMR | Rate | Mozambique | -3.024(-3.370,-2.678) |
| descending | 3 | 1990-2021 | ASMR | Rate | Solomon Islands | -2.841(-3.272,-2.411) |
| descending | 4 | 1990-2021 | ASMR | Rate | Rwanda | -2.805(-3.103,-2.508) |
| descending | 5 | 1990-2021 | ASMR | Rate | Burundi | -2.590(-2.847,-2.333) |
| ascending | 1 | 1990-2021 | ASDR | Rate | Central African Republic | 44.430(38.505,50.354) |
| ascending | 2 | 1990-2021 | ASDR | Rate | Benin | 33.501(24.128,42.874) |
| ascending | 3 | 1990-2021 | ASDR | Rate | South Sudan | 31.927(21.990,41.863) |
| ascending | 4 | 1990-2021 | ASDR | Rate | Republic of Nicaragua | 1.553(1.201,1.905) |
| ascending | 5 | 1990-2021 | ASDR | Rate | Bolivarian Republic of Venezuela | 1.022(0.496,1.547) |
| descending | 1 | 1990-2021 | ASDR | Rate | Europe | -0.012(-0.013,-0.011) |
| descending | 2 | 1990-2021 | ASDR | Rate | French Republic | -0.015(-0.025,-0.006) |
| descending | 3 | 1990-2021 | ASDR | Rate | Morocco | -0.025(-0.025,-0.024) |
| descending | 4 | 1990-2021 | ASDR | Rate | World Bank High Income | -0.030(-0.040,-0.020) |
| descending | 5 | 1990-2021 | ASDR | Rate | Republic of Costa Rica | -0.047(-0.072,-0.021) |
| High | 1 | 2021 | ASIR | Rate | Liberia | 27702.66(14565.45, 38887.49) |
| High | 2 | 2021 | ASIR | Rate | Benin | 27371.07(18769.46, 35280.94) |
| High | 3 | 2021 | ASIR | Rate | Burkina Faso | 26759.08(17858.4, 37140.73) |
| High | 4 | 2021 | ASIR | Rate | Solomon Islands | 26543.37(23807.76, 29509.52) |
| High | 5 | 2021 | ASIR | Rate | Sierra Leone | 25205.74(14430.05, 36137.61) |
| low | 1 | 2021 | ASIR | Rate | Saudi Arabia | 0.48(0.02, 2.25) |
| low | 2 | 2021 | ASIR | Rate | United Mexican States | 0.52(0.19, 1.05) |
| low | 3 | 2021 | ASIR | Rate | Republic of Korea | 0.76(0.19, 2.03) |
| low | 4 | 2021 | ASIR | Rate | Islamic Republic of Iran | 1.15(1.1, 1.17) |
| low | 5 | 2021 | ASIR | Rate | Socialist Republic of Viet Nam | 1.25(1.03, 1.48) |
| High | 1 | 2021 | ASPR | Rate | Liberia | 29248.89(13845.02, 47808.36) |
| High | 2 | 2021 | ASPR | Rate | Benin | 26381.29(18140.24, 35156.38) |
| High | 3 | 2021 | ASPR | Rate | Burkina Faso | 25118.45(15382.65, 36446.31) |
| High | 4 | 2021 | ASPR | Rate | Sierra Leone | 24551.27(11068.68, 42048.6) |
| High | 5 | 2021 | ASPR | Rate | Congo | 23674.27(17397.97, 32319.3) |
| low | 1 | 2021 | ASPR | Rate | Malaysia | 0.01(0.01, 0.02) |
| low | 2 | 2021 | ASPR | Rate | Saudi Arabia | 0.13(0.1, 0.16) |
| low | 3 | 2021 | ASPR | Rate | Dominican Republic | 2.4(2.03, 2.81) |
| low | 4 | 2021 | ASPR | Rate | Islamic Republic of Iran | 2.8(0.65, 11.64) |
| low | 5 | 2021 | ASPR | Rate | United Mexican States | 2.96(0.64, 15.69) |
| High | 1 | 2021 | ASMR | Rate | Burkina Faso | 173.13(75.38, 318.49) |
| High | 2 | 2021 | ASMR | Rate | Sierra Leone | 169.73(53.58, 334.2) |
| High | 3 | 2021 | ASMR | Rate | Niger | 140.04(44.76, 273.23) |
| High | 4 | 2021 | ASMR | Rate | Liberia | 136.29(43.99, 281.05) |
| High | 5 | 2021 | ASMR | Rate | Benin | 131.39(56.33, 255.11) |
| low | 1 | 2021 | ASMR | Rate | Oman | 0(0, 0) |
| low | 2 | 2021 | ASMR | Rate | Islamic Republic of Iran | 0(0, 0) |
| low | 3 | 2021 | ASMR | Rate | Saudi Arabia | 0(0, 0) |
| low | 4 | 2021 | ASMR | Rate | Kingdom of Thailand | 0(0, 0) |
| low | 5 | 2021 | ASMR | Rate | United Mexican States | 0(0, 0) |
| High | 1 | 2021 | ASDR | Rate | Sierra Leone | 8940.31(3029.75, 17358.47) |
| High | 2 | 2021 | ASDR | Rate | Burkina Faso | 8938.18(4195.32, 15779.33) |
| High | 3 | 2021 | ASDR | Rate | Niger | 7344.92(2624.86, 13447.18) |
| High | 4 | 2021 | ASDR | Rate | Benin | 6891.46(3261.99, 12411.94) |
| High | 5 | 2021 | ASDR | Rate | Liberia | 6836.6(2425.73, 13296.02) |
| low | 1 | 2021 | ASDR | Rate | Republic of Guatemala | 0.01(0, 0.01) |
| low | 2 | 2021 | ASDR | Rate | Malaysia | 0.05(0.03, 0.07) |
| low | 3 | 2021 | ASDR | Rate | United Mexican States | 0.08(0.07, 0.1) |
| low | 4 | 2021 | ASDR | Rate | Republic of Korea | 0.1(0.08, 0.13) |
| low | 5 | 2021 | ASDR | Rate | Republic of Costa Rica | 0.26(0.21, 0.32) |
| High | 1 | 2021 | incidence | number | Nigeria | 71275231(50133632, 92032177) |
| High | 2 | 2021 | incidence | number | Congo | 26756910(20405699, 34661135) |
| High | 3 | 2021 | incidence | number | Uganda | 10269172(7709280, 12875208) |
| High | 4 | 2021 | incidence | number | Mozambique | 9958909(7037360, 13088080) |
| High | 5 | 2021 | incidence | number | Burkina Faso | 8594162(5419217, 12383140) |
| low | 1 | 2021 | incidence | number | Cabo Verde | 51(43, 60) |
| low | 2 | 2021 | incidence | number | Bhutan | 64(56, 75) |
| low | 3 | 2021 | incidence | number | Oman | 121(1, 898) |
| low | 4 | 2021 | incidence | number | Saudi Arabia | 199(10, 928) |
| low | 5 | 2021 | incidence | number | Republic of Costa Rica | 357(357, 357) |
| High | 1 | 2021 | prevalence | number | Nigeria | 50013813(35765803, 61733841) |
| High | 2 | 2021 | prevalence | number | Democratic Republic of the Congo | 23050050(16935623, 31478579) |
| High | 3 | 2021 | prevalence | number | Uganda | 8142805(6505638, 10112367) |
| High | 4 | 2021 | prevalence | number | Mozambique | 7163269(4736144, 9736820) |
| High | 5 | 2021 | prevalence | number | Burkina Faso | 6249181(3828388, 9060416) |
| low | 1 | 2021 | prevalence | number | Republic of Guatemala | 2(1, 3) |
| low | 2 | 2021 | prevalence | number | Malaysia | 43(34, 55) |
| low | 3 | 2021 | prevalence | number | Dominican Republic | 310(73, 1279) |
| low | 4 | 2021 | prevalence | number | Cabo Verde | 364(326, 404) |
| low | 5 | 2021 | prevalence | number | Eswatini | 485(130, 618) |
| High | 1 | 2021 | death | number | Nigeria | 227529(74960, 475655) |
| High | 2 | 2021 | death | number | Congo | 62359(27976, 112100) |
| High | 3 | 2021 | death | number | Uganda | 45361(14826, 82604) |
| High | 4 | 2021 | death | number | Burkina Faso | 35963(16365, 64139) |
| High | 5 | 2021 | death | number | Niger | 33664(11795, 61380) |
| low | 1 | 2021 | death | number | Oman | 0(0, 0) |
| low | 2 | 2021 | death | number | Cabo Verde | 0(0, 0) |
| low | 3 | 2021 | death | number | Bhutan | 0(0, 0) |
| low | 4 | 2021 | death | number | Republic of Panama | 0(0, 1) |
| low | 5 | 2021 | death | number | Islamic Republic of Iran | 0(0, 1) |
| High | 1 | 2021 | DALY | number | Nigeria | 16497677(6034840, 32747630) |
| High | 2 | 2021 | DALY | number | Congo | 5236328(2581070, 8931619) |
| High | 3 | 2021 | DALY | number | Uganda | 3658759(1288958, 6372019) |
| High | 4 | 2021 | DALY | number | Burkina Faso | 2570349(1233546, 4419194) |
| High | 5 | 2021 | DALY | number | Niger | 2549576(956590, 4503771) |
| low | 1 | 2021 | DALY | number | Republic of Guatemala | 1(0, 1) |
| low | 2 | 2021 | DALY | number | Republic of Costa Rica | 13(11, 15) |
| low | 3 | 2021 | DALY | number | Republic of Suriname | 16(5, 36) |
| low | 4 | 2021 | DALY | number | Malaysia | 18(11, 25) |
| low | 5 | 2021 | DALY | number | Bhutan | 21(15, 29) |

Notes: Between 1990 and 2021, 101 countries reported ASIR, ASPR, ASMR, and ASDR for total leishmaniasis. Abbreviations: AAPC, average annual percent change; ASIR, age-standardized incidence rate; ASDR, age-standardized disability-adjusted life years; ASMR, age-standardized mortality rate; ASPR, age-standardized prevalence rate; DALYs, disability-adjusted life years; GBD, Global Burden of Disease; *UI*, uncertainty interval.

**
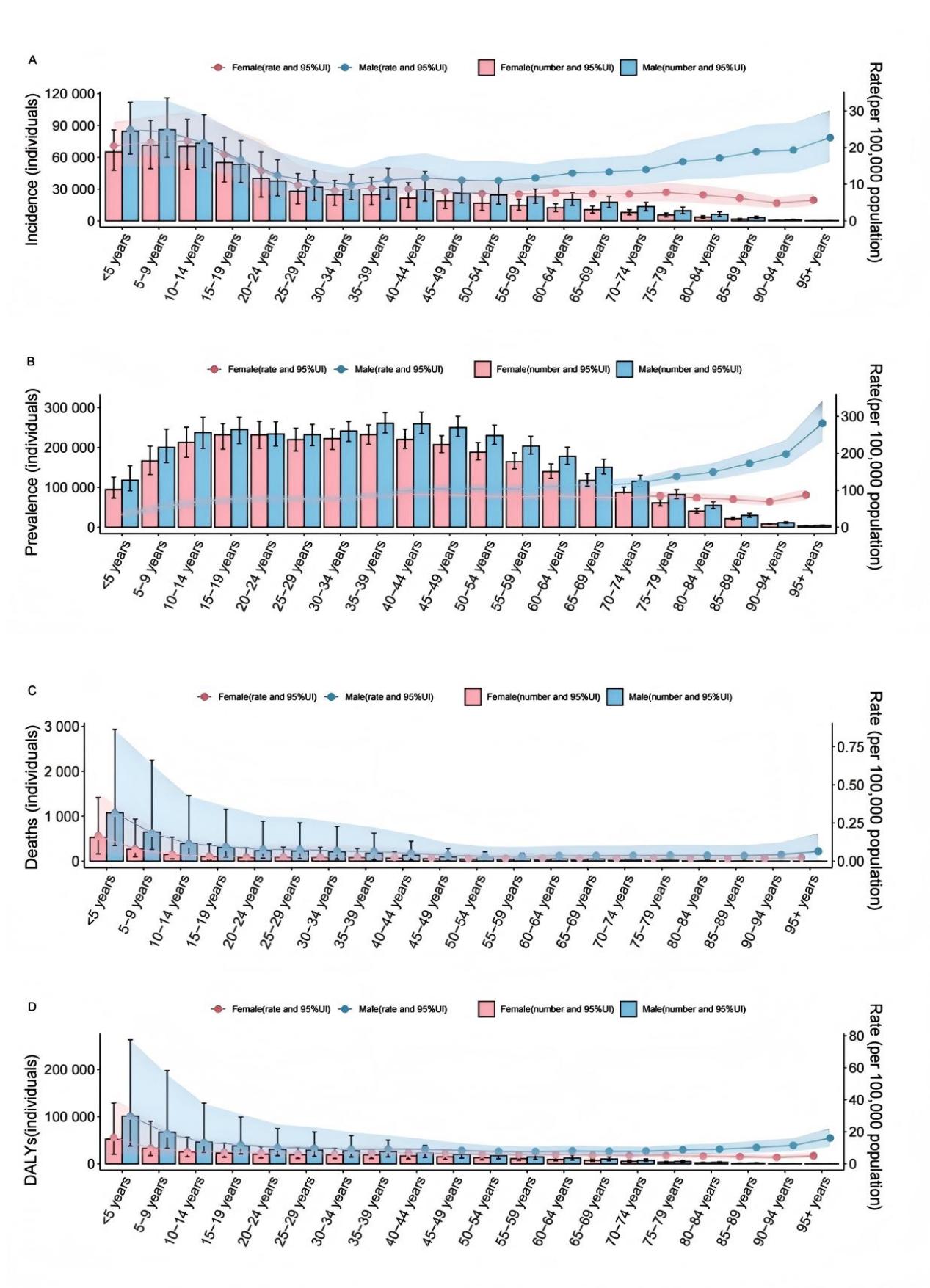
**

**Fig. S1.** The specific rate of leishmaniasis showed notable differences across age and gender distributions in 2021 year (A: incidence rate, B: prevalence, C: mortality rate, D: DALY rate). Abbreviations: DALYs, disability-adjusted life years; *UI*, uncertainty interval.

**Table S6.** The association between the burden of leishmaniasis and the SDI in global.

| Locations | Index | *r* | *P* |
| --- | --- | --- | --- |
| 204 countries and territories, 2021 | ASIR | -0.3032 | <0.001 |
|  | ASPR | -0.2964 | <0.001 |
|  | ASMR | -0.2627 | <0.001 |
|  | ASDR | -0.3329 | <0.001 |
| 204 countries and territories, 2021 | Incident cases | -0.3233 | <0.001 |
|  | Prevalent cases | -0.2995 | <0.001 |
|  | Deaths | -0.2947 | <0.001 |
|  | DALYs cases | -0.3521 | <0.001 |
| Global+21 geographic regions, 1990–2021 | ASIR | -0.4504 | <0.001 |
|  | ASPR | -0.4315 | <0.001 |
|  | ASMR | -0.5114 | <0.001 |
|  | ASDR | -0.556 | <0.001 |
| Global+21 geographic regions, 1990–2021 | Incident cases | -0.4948 | <0.001 |
|  | Prevalent cases | -0.4465 | <0.001 |
|  | Deaths | -0.4882 | <0.001 |
|  | DALYs cases | -0.5299 | <0.001 |

Notes: Between 1990 and 2021, no data on the incidence rate of leishmaniasis have been reported from Oceania, Eastern Europe, the high-income Asia Pacific, or Australasia. In addition, 101 countries reported ASIR, ASPR, ASMR, and ASDR for total leishmaniasis. Abbreviations: AAPC, average annual percent change; ASIR, age-standardized incidence rate; ASDR, age-standardized disability-adjusted life years; ASMR, age-standardized mortality rate; ASPR, age-standardized prevalence rate; DALYs, disability-adjusted life years; GBD, Global Burden of Disease; SDI, sociodemographic index; *UI*, uncertainty interval.

**Table S7.** Based on the BAPC model, the trends in the disease burden indicators for leishmaniasis from 2022 to 2035 were predicted in global and 21 GBD geographical regions.

| Location | Index | Value (95% *CI*), 2035 | AAPC (95% *CI*) | EAPC (95% *CI*) |
| --- | --- | --- | --- | --- |
| Global | ASIR | 11.58(6.16, 16.99) | -1.13 (-1.14, -1.12) | -1.13(-1.15, -1.11) |
| East Asia | ASIR | 0.06(0.02, 0.10) | -6.82 (-6.83, -6.80) | -6.82(-6.86, -6.78) |
| Southeast Asia | ASIR | 2.61(0.00, 5.67) | 5.98 (5.98, 5.98) | 5.98(5.97, 5.98) |
| Oceania | ASIR | 0.01(0.00, 0.01) | -0.00 (-0.00, -0.00) | -0.00(-0.00, -0.00) |
| Central Asia | ASIR | 3.9(0.06, 7.74) | -4.16 (-4.16, -4.16) | -4.16(-4.16, -4.16) |
| Central Europe | ASIR | 0.04(0.01, 0.07) | -8.75 (-8.76, -8.74) | -8.74(-8.76, -8.72) |
| Eastern Europe | ASIR | NA | NA | NA |
| High-income Asia Pacific | ASIR | NA | NA | NA |
| Australasia | ASIR | NA | NA | NA |
| Western Europe | ASIR | 0.23(0.15, 0.3) | -2.02 (-2.04, -2.01) | -2.02(-2.05, -2.00) |
| Southern Latin America | ASIR | 1.66(0.63, 2.69) | -0.48 (-0.49, -0.48) | -0.48(-0.49, -0.48) |
| High-income North America | ASIR | 0.04(0.03, 0.06) | 2.22 (2.22, 2.22) | 2.22(2.22, 2.22) |
| Caribbean | ASIR | 2.99(1.08, 4.91) | -2.12 (-2.13, -2.12) | -2.12(-2.13, -2.12) |
| Andean Latin America | ASIR | 48.48(33.63, 63.33) | -1.72 (-1.73, -1.72) | -1.73(-1.73, -1.72) |
| Central Latin America | ASIR | 22.29(4.09, 40.49) | -1.41 (-1.41, -1.41) | -1.41(-1.42, -1.40) |
| Tropical Latin America | ASIR | 24.59(13.53, 35.65) | -1.04 (-1.05, -1.03) | -1.04(-1.06, -1.02) |
| North Africa and Middle East | ASIR | 148.44(79.33, 217.56) | 0.87 (0.87, 0.88) | 0.87(0.86, 0.89) |
| South Asia | ASIR | 1.26(0.43, 2.1) | -6.77 (-6.78, -6.77) | -6.76(-6.78, -6.75) |
| Central Sub-Saharan Africa | ASIR | 1.22(0.00, 3.1) | -7.38 (-7.38, -7.37) | -7.38(-7.40, -7.36) |
| Eastern Sub-Saharan Africa | ASIR | 0.75(0.00, 1.49) | -9.93 (-9.94, -9.92) | -9.93(-9.95, -9.91) |
| Southern Sub-Saharan Africa | ASIR | 0.07(0.05, 0.1) | 0.02 (0.02, 0.02) | 0.02(0.02, 0.02) |
| Western Sub-Saharan Africa | ASIR | 3.84(1.73, 5.95) | 0.52 (0.49, 0.54) | 0.53(0.48, 0.57) |
| Global | ASPR | 85.78(79.31, 92.25) | 0.86 (0.85, 0.86) | 0.86(0.84, 0.87) |
| East Asia | ASPR | 0.64(0.56, 0.72) | -3.50 (-3.54, -3.46) | -3.49(-3.54, -3.44) |
| Southeast Asia | ASPR | 4(2.65, 5.34) | 4.75 (4.67, 4.82) | 4.75(4.59, 4.91) |
| Oceania | ASPR | NA | NA | NA |
| Central Asia | ASPR | 78.28(66.86, 89.7) | -2.61 (-2.62, -2.60) | -2.61(-2.63, -2.60) |
| Central Europe | ASPR | 0.39(0.35, 0.43) | -3.38 (-3.40, -3.36) | -3.38(-3.43, -3.34) |
| Eastern Europe | ASPR | NA | NA | NA |
| High-income Asia Pacific | ASPR | NA | NA | NA |
| Australasia | ASPR | NA | NA | NA |
| Western Europe | ASPR | 0.66(0.59, 0.72) | -0.87 (-0.87, -0.87) | -0.87(-0.87, -0.87) |
| Southern Latin America | ASPR | 13.08(10.75, 15.4) | 0.09 (0.09, 0.10) | 0.10(0.09, 0.10) |
| High-income North America | ASPR | 0.11(0.09, 0.12) | 0.76 (0.75, 0.77) | 0.76(0.74, 0.78) |
| Caribbean | ASPR | 51.82(47.54, 56.09) | -0.15 (-0.15, -0.14) | -0.14(-0.15, -0.14) |
| Andean Latin America | ASPR | 509.66(455.86, 563.47) | -0.57 (-0.59, -0.55) | -0.57(-0.60, -0.54) |
| Central Latin America | ASPR | 203.76(183.08, 224.43) | -0.43 (-0.44, -0.43) | -0.43(-0.44, -0.42) |
| Tropical Latin America | ASPR | 271.07(239.05, 303.08) | -0.71 (-0.73, -0.69) | -0.70(-0.75, -0.65) |
| North Africa and Middle East | ASPR | 773.87(706.69, 841.05) | 1.25 (1.21, 1.29) | 1.25(1.18, 1.32) |
| South Asia | ASPR | 18.01(14.91, 21.1) | -0.17 (-0.19, -0.16) | -0.18(-0.20, -0.15) |
| Central Sub-Saharan Africa | ASPR | 12.17(8.02, 16.32) | -1.99 (-2.00, -1.98) | -1.99(-2.01, -1.97) |
| Eastern Sub-Saharan Africa | ASPR | 7.07(4.55, 9.6) | -3.13 (-3.14, -3.11) | -3.13(-3.16, -3.10) |
| Southern Sub-Saharan Africa | ASPR | 0.62(0.55, 0.69) | -0.18 (-0.19, -0.18) | -0.18(-0.19, -0.17) |
| Western Sub-Saharan Africa | ASPR | 45.8(37.58, 54.02) | 0.57 (0.57, 0.57) | 0.57(0.57, 0.58) |
| Global | ASMR | 0.02(0, 0.04) | -8.74 (-8.75, -8.74) | -8.75(-8.76, -8.73) |
| East Asia | ASMR | 0(0, 0.01) | -6.74 (-6.75, -6.73) | -6.74(-6.76, -6.72) |
| Southeast Asia | ASMR | 0(0, 0) | -14.30 (-14.30, -14.29) | -14.30(-14.32, -14.29) |
| Oceania | ASMR | NA | NA | NA |
| Central Asia | ASMR | 0.05(0.01, 0.09) | 1.68 (1.67, 1.69) | 1.68(1.66, 1.71) |
| Central Europe | ASMR | 0(0, 0) | -12.72 (-12.74, -12.70) | -12.72(-12.76, -12.68) |
| Eastern Europe | ASMR | NA | NA | NA |
| High-income Asia Pacific | ASMR | NA | NA | NA |
| Australasia | ASMR | NA | NA | NA |
| Western Europe | ASMR | 0(0, 0.01) | -4.56 (-4.56, -4.55) | -4.56(-4.57, -4.55) |
| Southern Latin America | ASMR | 0(0, 0) | -6.09 (-6.09, -6.08) | -6.09(-6.09, -6.08) |
| High-income North America | ASMR | 0(0, 0) | 0.00 (0.00, 0.00) | 0.00(0.00, 0.00) |
| Caribbean | ASMR | 0(0, 0) | -16.40 (-16.40, -16.39) | -16.40(-16.40, -16.39) |
| Andean Latin America | ASMR | 0(0, 0.01) | -4.66 (-4.66, -4.66) | -4.66(-4.66, -4.65) |
| Central Latin America | ASMR | 0(0, 0.01) | -3.74 (-3.74, -3.74) | -3.74(-3.75, -3.73) |
| Tropical Latin America | ASMR | 0.41(0.22, 0.6) | -1.55 (-1.56, -1.55) | -1.55(-1.57, -1.54) |
| North Africa and Middle East | ASMR | 0.02(0.01, 0.03) | -8.45 (-8.45, -8.45) | -8.45(-8.45, -8.45) |
| South Asia | ASMR | 0.02(0, 0.03) | -10.30 (-10.30, -10.29) | -10.30(-10.31, -10.29) |
| Central Sub-Saharan Africa | ASMR | 0.11(0.00, 0.33) | -8.53 (-8.54, -8.53) | -8.53(-8.55, -8.52) |
| Eastern Sub-Saharan Africa | ASMR | 0.07(0.00, 0.16) | -10.12 (-10.12, -10.12) | -10.12(-10.12, -10.12) |
| Southern Sub-Saharan Africa | ASMR | NA | NA | NA |
| Western Sub-Saharan Africa | ASMR | 0.07(-0.09, 0.23) | 4.36 (4.36, 4.37) | 4.36(4.35, 4.38) |
| Global | ASDR | 3.24(1.48, 5.01) | -7.68 (-7.69, -7.67) | -7.68(-7.69, -7.66) |
| East Asia | ASDR | 0.31(0.17, 0.45) | -6.81 (-6.82, -6.80) | -6.81(-6.83, -6.79) |
| Southeast Asia | ASDR | 0.12(0.05, 0.19) | -1.58 (-1.60, -1.56) | -1.57(-1.61, -1.54) |
| Oceania | ASDR | NA | NA | NA |
| Central Asia | ASDR | 7.56(6.15, 8.96) | -1.48 (-1.49, -1.47) | -1.48(-1.49, -1.46) |
| Central Europe | ASDR | 0.11(0.02, 0.19) | -9.68 (-9.69, -9.67) | -9.68(-9.70, -9.66) |
| Eastern Europe | ASDR | NA | NA | NA |
| High-income Asia Pacific | ASDR | NA | NA | NA |
| Australasia | ASDR | NA | NA | NA |
| Western Europe | ASDR | 0.26(0.03, 0.49) | -4.31 (-4.32, -4.31) | -4.31(-4.31, -4.30) |
| Southern Latin America | ASDR | 0.73(0.57, 0.89) | -1.58 (-1.59, -1.57) | -1.58(-1.60, -1.55) |
| High-income North America | ASDR | 0.01(0, 0.01) | 0.34 (0.33, 0.35) | 0.34(0.32, 0.36) |
| Caribbean | ASDR | 3.07(2.78, 3.37) | -0.58 (-0.58, -0.57) | -0.58(-0.59, -0.56) |
| Andean Latin America | ASDR | 28.14(24.75, 31.54) | -1.60 (-1.61, -1.58) | -1.60(-1.63, -1.57) |
| Central Latin America | ASDR | 12.22(11.15, 13.28) | -1.01 (-1.02, -1.00) | -1.01(-1.03, -0.99) |
| Tropical Latin America | ASDR | 42.5(30.99, 54.01) | -1.53 (-1.54, -1.51) | -1.53(-1.55, -1.50) |
| North Africa and Middle East | ASDR | 26.45(20.84, 32.05) | -3.54 (-3.55, -3.53) | -3.54(-3.56, -3.52) |
| South Asia | ASDR | 1.15(0.09, 2.21) | -11.11 (-11.12,-11.10) | -11.11(-11.13, -11.09) |
| Central Sub-Saharan Africa | ASDR | 7.28(0.00, 21.93) | -8.95 (-8.96, -8.93) | -8.95(-8.98, -8.92) |
| Eastern Sub-Saharan Africa | ASDR | 5.06(0.00, 11.7) | -10.32 (-10.33, -10.32) | -10.33(-10.34, -10.31) |
| Southern Sub-Saharan Africa | ASDR | 0.04(0.03, 0.05) | -0.19 (-0.19, -0.19) | -0.19(-0.19, -0.19) |
| Western Sub-Saharan Africa | ASDR | 18.45(8.61, 28.29) | 8.62 (8.50, 8.73) | 8.62(8.39, 8.86) |

Notes: Between 1990 and 2021, no data on the incidence rate of leishmaniasis have been reported from Oceania, Eastern Europe, the high-income Asia Pacific, or Australasia. Therefore, the four disease burden indicators for leishmaniasis in these areas remain unpredictable. Abbreviations: AAPC, average annual percent change; ASIR, age-standardized incidence rate; ASDR, age-standardized disability-adjusted life years; ASMR, age-standardized mortality rate; ASPR, age-standardized prevalence rate; BAPC, Bayesian age-period-cohort; *CI*, confidence interval; DALYs, disability-adjusted life years; EAPC, estimated annual percentage change; GBD, Global Burden of Disease.
